# Inhibition of Parkinson’s disease–related LRRK2 by type I and type II kinase inhibitors: Activity and structures

**DOI:** 10.1126/sciadv.adk6191

**Published:** 2023-12-01

**Authors:** Marta Sanz Murillo, Amalia Villagran Suarez, Verena Dederer, Deep Chatterjee, Jaime Alegrio Louro, Stefan Knapp, Sebastian Mathea, Andres E. Leschziner

**Affiliations:** ^1^ Department of Cellular and Molecular Medicine, School of Medicine, University of California San Diego, La Jolla, CA 92093, USA.; ^2^ Aligning Science Across Parkinson’s (ASAP) Collaborative Researcg Network, Chevy Chase, MD 20815, USA..; ^3^ Institute of Pharmaceutical Chemistry, Goethe-Universität, Frankfurt 60438, Germany.; ^4^ Structural Genomics Consortium (SGC), Buchmann Institute for Life Sciences, Goethe-Universität, Frankfurt 60438, Germany.; ^5^ Department of Molecular Biology, School of Biological Sciences, University of California San Diego, La Jolla, CA 92093, USA.

## Abstract

Mutations in leucine-rich repeat kinase 2 (LRRK2) are a common cause of familial Parkinson’s disease (PD) and a risk factor for the sporadic form. Increased kinase activity was shown in patients with both familial and sporadic PD, making LRRK2 kinase inhibitors a major focus of drug development efforts. Although much progress has been made in understanding the structural biology of LRRK2, there are no available structures of LRRK2 inhibitor complexes. To this end, we solved cryo–electron microscopy structures of LRRK2, wild-type and PD-linked mutants, bound to the LRRK2-specific type I inhibitor MLi-2 and the broad-spectrum type II inhibitor GZD-824. Our structures revealed an active-like LRRK2 kinase in the type I inhibitor complex, and an inactive DYG-out in the type II inhibitor complex. Our structural analysis also showed how inhibitor-induced conformational changes in LRRK2 are affected by its autoinhibitory N-terminal repeats. The structures provide a template for the rational development of LRRK2 kinase inhibitors covering both canonical inhibitor binding modes.

## Teaser

Structures of Parkinson’s Disease-linked LRRK2 bound to inhibitors provide insights for the design of small molecule therapeutics.

## INTRODUCTION

Mutations in leucine-rich repeat kinase 2 (LRRK2) are one of the most common drivers of the familial form of Parkinson’s disease (PD) (*1*–*3*) and are also associated with increased risk for the sporadic form of PD (*1*, *4*). The most frequent PD-linked mutations in LRRK2 increase its kinase activity (*5*–*8*), but hyperactivation of an otherwise wild-type (WT) LRRK2 has also been reported in idiopathic PD (*9*). As a result, LRRK2 has become a major target for the development of kinase inhibitors as therapeutics for PD (*10*, *11*).

**Fig. 1.**
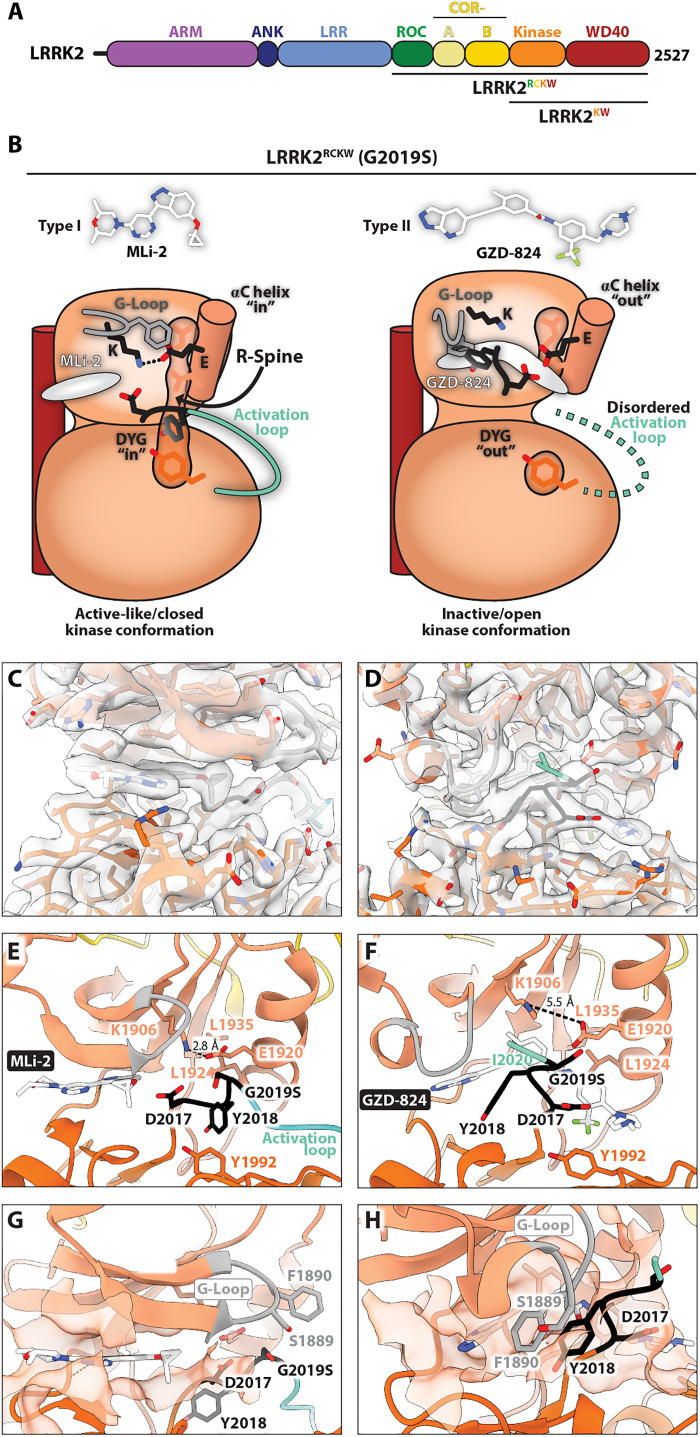
LRRK2^RCKW^(G2019S) bound to MLi-2 and GZD-824. (**A**) Domain architecture of LRRK2. The color coding of domains is used throughout this work. The three constructs used in this study—LRRK2, LRRK2^RCKW^, and LRRK2^KW^—are indicated. (**B**) Cartoon summary of the main features of the structures presented here. The inhibitors used, the type I MLi-2 and type II GZD-824, are shown in stick representation. All panels below correspond to the structure depicted in cartoon form here. (**C** and **D**) Cryo–electron microscopy (cryo-EM) maps and models for the inhibitor binding site and surrounding regions for MLi-2 (C) and GZD-824 (D) bound to LRRK2^RCKW^(G2019S). (**E** and **F**) Close-up of the inhibitor binding site and kinase active site for the same structures shown in (C) and (D). Major features (G-loop, Lys^1906^-Glu^192^^0^ interaction, DYG motif, R-spine, and activation loop) are indicated. (**G** and **H**) Inhibitor binding pocket. The semitransparent orange surface indicates the solvent-accessible surface for those residues in contact with the inhibitor. Some of the features highlighted in (E) and (F) are shown here as well.

LRRK2 is a large, 2527-residue multidomain protein. Its N-terminal half is composed of armadillo (ARM), ankyrin (ANK), and leucine-rich (LRR) repeat domains, while its C-terminal half contains two catalytic domains, a guanosine triphosphatase (GTPase) (Ras of complex or ROC) and a Ser/Thr kinase, as well as two architectural domains, a C-terminal of ROC (COR) between the GTPase and the kinase and a WD40 domain at the end (Fig. 1A). Recent studies using cryo–electron microscopy (cryo-EM) have described structures for the C-terminal half of LRRK2 (“LRRK2^RCKW^”) (*12*), full-length LRRK2 alone (*13*) and bound to Rab29 (*14*), and both LRRK2 (*15*) and LRRK2^RCKW^ (*16*) bound to microtubules. Although a recent study proposed structures of LRRK2^RCKW^ bound to inhibitors generated using molecular dynamics (*17*), there are no published experimental structures of inhibitor-bound LRRK2, an important gap in our understanding of this protein as a therapeutic target.

### LRRK2 kinase inhibitors

Small molecules of diverse chemotypes have been developed as type I kinase inhibitors for LRRK2 (fig. S1A). The first inhibitor to be introduced with an acceptable selectivity profile was LRRK2-IN-1 (*18*). However, its use for in vivo applications has been limited because of its low brain penetrance. HG-10-102-01, in contrast, was developed as a brain penetrant inhibitor (*19*), and its amino-pyrimidine–based scaffold recently experienced a renaissance as a proteolysis targeting chimera warhead (*20*). MLi-2, an inhibitor with high affinity and a superb selectivity profile, has been important for the field (*21*). Ever since its introduction in 2015, it has been regarded as the gold standard LRRK2 inhibitor and this chemical probe has been used in numerous studies, including clinical trials. Another powerful but less prominent inhibitor is PFE-360 (*22*). Like MLi-2, its affinity for LRRK2 and its selectivity profile are both excellent. The last entry in our incomplete list is the type I inhibitor DNL201 (developed as GNE0877) (*23*). This compound has been shown to efficiently inhibit LRRK2 in patients with PD (*24*), and the related molecule DNL151 (renamed BIIB122) has currently entered phase 2b clinical testing (clinicaltrials.gov).

Less effort has been undertaken to develop type II inhibitors for the LRRK2 kinase domain. To date, no selective type II inhibitors have been published (*25*). However, several type II inhibitors with a broad kinase target spectrum have been shown to bind to LRRK2's kinase with high affinity, namely, ponatinib, GZD-824 (*12*), and rebastinib (*26*). However, their numerous kinase off-targets prevent their application as chemical tools or for the treatment of PD.

The need for LRRK2 selective type II inhibitors is not limited to their therapeutic potential. The recent structural studies of LRRK2 listed above (*12*–*16*) have made it clear that conformational changes in LRRK2 driven by its kinase are likely to play central roles in its activation and subcellular localization. Having reagents that stabilize the LRRK2 kinase domain in either a closed, active-like conformation (type I inhibitors) or in its open, inactive conformation (type II inhibitors) would have a major impact on our ability to dissect its catalytic and scaffolding functions in cells and in vivo.

Here, we set out to bridge the gap in our understanding of the structural biology of LRRK2 inhibition. We present cryo-EM structures of LRRK2^RCKW^, WT, and carrying the PD-linked mutations G2019S and I2020T, bound to the LRRK2-specific type I inhibitor MLi-2 or the broad-spectrum type II inhibitor GZD-824. We also present structures of full-length LRRK2 bound to these inhibitors to understand how the presence of the N-terminal repeats in LRRK2 affects inhibitor-induced conformational changes in the context of the full-length protein. The work presented here should help in the design of the next generation of LRRK2-specific inhibitors.

## RESULTS

### Inhibitor binding stabilizes the LRRK2 kinase domain

The tight binding of small molecules to a protein usually stabilizes the protein and increases the temperature at which it denatures. We characterized the binding of diverse type I and type II inhibitors (Table 1 and fig. S1A) by measuring the thermostability increase of a truncated LRRK2 protein consisting of its kinase and WD40 domains (“LRRK2^KW^”) (Fig. 1A) in the presence of inhibitors using differential scanning fluorimetry (DSF). The melting profile showed two clearly separated transitions most likely corresponding to the two domains, the kinase and the WD domain. In agreement with this hypothesis, only the melting curve of one of the phase transitions shifted in the presence of the diverse inhibitors tested suggesting that the transition at lower melting temperature corresponds to the kinase domain. However, in the absence of inhibitors, LRRK2^KW^ was relatively unstable compared to other kinases [melting temperature (*T*_M_) of 39°C] (*27*). Both type I and type II inhibitors stabilized LRRK2^KW^ substantially, with *T*_M_ shifts of up to 20°C, indicating strong binding to the kinase domain, while the melting curve of the WD domain remained unaffected (fig. S1, B and D).

**Table 1.** Kinase inhibitors tested in this study Inhibitors are listed along with their simplified molecular-input line-entry system (SMILES) formulae, vendor, and stock numbers.

Inhibitor	SMILES	Vendor	Order no.
MLi-2	C[C@@H]1CN(C[C@@H](O1)C)C2 = NC=NC(=C2)C3 = NNC4 = C3C=C(C=C4)OC5(CC5)C	Tocris Bioscience	1627091-47-7/5756
PF-360	N#CC1 = CC(C2 = CNC3 = NC=NC(N4CCOCC4) = C32) = CN1C	MedChem Express	1527475-61-1/HY-120085
HG-10-102-01	COC1 = CC(C(N2CCOCC2) = O) = CC=C1NC3 = NC(NC) = C(Cl)C=N3	MedChem Express	1351758-81-0/HY-13488
LRRK2-IN-1	O=C(C1 = CC(OC) = C(NC2 = NC=C3C(N(C4 = C(C(N3C) = O)C=CC=C4)C) = N2)C=C1)N5CCC(CC5)N6CCN(CC6)C	Merck Millipore	1234480-84-2/438193
DNL201	CC1 = NN(C=C1NC2 = NC=C(C(=N2)NC)C(F)(F)F)C(C)(C)C#N	MedChem Express	1374828-69-9
Rebastinib	CC(C)(C)C1 = NN(C(NC(NC2 = C(F)C=C(OC3 = CC(C(NC) = O) = NC=C3)C=C2) = O) = C1)C4 = CC=C5C(C=CC=N5) = C4	MedChem Express	1020172-07-9/HY-13024
Ponatinib	CN(CC1)CCN1CC2 = C(C(F)(F)F)C=C(NC(C3 = CC=C(C)C(C#CC4 = CN=C5N4N=CC=C5) = C3) = O)C=C2	MedChem Express	943319-70-8/HY-12047
GZD-824	O=C(NC1 = CC=C(CN2CCN(C)CC2)C(C(F)(F)F) = C1)C3 = CC=C(C)C(C#CC4 = CN=C(NN=C5)C5 = C4) = C3	MedChem Express	1257628-77-5/HY-15666

### A mass spectrometry–based assay to monitor LRRK2 activity and inhibition

Increased kinase catalytic activity and impaired autoinhibition are features of LRRK2 PD variants (*5*–*8*). To compare the activity of the selected LRRK2 inhibitors, we developed a mass spectrometry (MS)–based kinase activity assay using an established endogenous substrate. We subjected the Rab8A GTPase domain (residues 6 to 176) to LRRK2^RCKW^ phosphorylation in the presence of varying concentrations of a given inhibitor and directly quantified the amount of phosphorylated Rab8A (pRab8A) by ESI-MS (fig. S1C). Most type I and type II inhibitors inhibited the phosphorylation reaction with IC_50_ (median inhibitory concentration) values between 7 and 50 nM. Unexpectedly, the inhibitors HG-10-102-01 and LRRK2-IN-1 stood out with IC_50_ values in the high nanomolar range. Together, there was a good correlation between the thermal shift data and the IC_50_ values (fig. S1D).

### Cryo-EM structures of LRRK2^RCKW^(G2019S) bound to type I and type II inhibitors

To understand how inhibitors of the two main canonical types interact with the catalytic domain of LRRK2, we began by determining cryo-EM structures of LRRK2^RCKW^, a well-characterized construct consisting of the C-terminal half of LRRK2 containing all catalytic domains (Fig. 1A) (*12*, *16*, *17*, *26*), bound to the LRRK2-specific inhibitor MLi-2 (type I) or the broad-spectrum GZD-824 (type II), in the presence of guanosine diphosphate (GDP) as a ligand for the GTPase (ROC) domain. We used a variant of LRRK2^RCKW^ carrying the G2019S mutation, the most common mutation associated with PD (*28*) and one of two, along with I2020T, located in the kinase domain (Fig. 1, figs. S3 to S5, table S1, and movie S1).

We solved these structures, as well as all additional cryo-EM structures reported here, bound to an LRRK2-specific designed ankyrin repeat protein (DARPin) (*29*) that we refer to as “E11.” This DARPin was developed screening LRRK2^RCKW^, and it is tightly bound to its WD40 domain (*30*). We have found that adding the E11 DARPin to our cryo-EM samples consistently leads to structures with better resolution, most likely due to a reduction in the preferred orientation that LRRK2 and LRRK2^RCKW^ tend to adopt on cryo-EM grids, although we cannot rule out a contribution from their additional mass (*30*). For simplicity, we will omit “E11” from the names of the complexes discussed in this work. The resolutions of our maps were sufficient to reveal the molecular details of the kinase active site. The MLi-2 map had an overall resolution of 2.74 Å and local resolutions as high as 2.4 Å around the kinase’s active site (fig. S3 and table S1). The GZD-824 map had an overall resolution of 3.11 Å and local resolutions as high as 2.7 Å around the kinase’s active site after focused refinement (fig. S4 and table S1).

Figure 1B and movie S1 summarize the main features of the two structures. MLi-2 bound as expected in the adenosine 5′-triphosphate (ATP)–binding pocket of LRRK2’s kinase, with its indazole group making hydrogen bonds with the backbones of E1948 and A1950 in the kinase hinge region, as recently proposed based on molecular dynamics simulations (*17*). In contrast to those simulations, however, the glycine-rich (G) loop was extended in our structure, as expected for an active kinase domain, and F1890, located at the tip of the G-loop, was not involved in coordinating MLi-2. Also as expected, MLi-2 binding stabilized the DYG “in” and αC “in” conformation, a fully formed R-spine, and an ordered activation loop (Fig. 1, B, C, and E). (Note: even though we will continue using the “DYG” nomenclature throughout the paper, DYG is DYS in the G2019S mutant.) We observed an inactive conformation with DYG “out”, αC “out”, a broken R-spine, and a disordered activation loop in the presence of GZD-824 (Fig. 1, B, D, and F). The binding of GZD-824 to the ATP-binding pocket was shifted away from the hinge region relative to MLi-2; its pyrazolopyridine group partially overlapped with the location of the pyrimidine and indazole groups of MLi-2, with the rest of the molecule extending toward and past the αC helix. As expected from a larger inhibitor, the binding pocket for GZD-824 was enlarged by the DYG-out movement and opening of the allosteric back pocket, involving all the main components of the kinase’s active site (Fig. 1H). A major difference between the two structures was the conformation of their G-loops (Fig. 1, G and H). While the G-loop was in an extended conformation in LRRK2^RCKW^(G2019S):MLi-2 (Fig. 1G), it was sharply bent in LRRK2^RCKW^(G2019S):GZD-824, with F1890 interacting with the pyrazolopyridine group in GZD-824 (Fig. 1H). In turn, Y2018 from the DYG motif interacted with F1890 (Fig. 1H).

### Structural basis for decreased affinity of MLi-2 for LRRK2(G2019S)

**Fig. 2.**
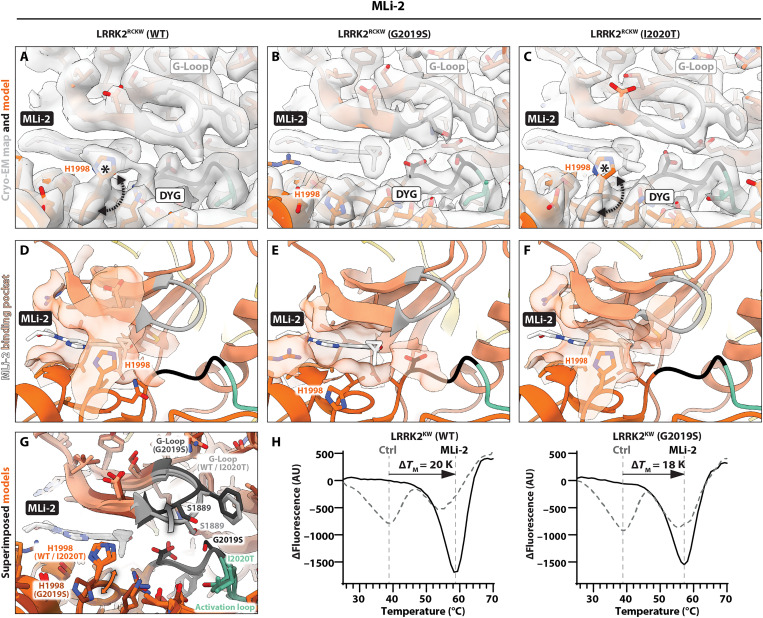
Binding of MLi-2 to LRRK2^RCKW^ WT, G2019S, and I2020T. (**A** to **C**) Cryo-EM maps and models for the MLi-2 binding site and surrounding regions for LRRK2^RCKW^ WT (A), G2019S (B), and I2020T (C). The asterisks in (A) and (C) highlight densities present in the WT and I2020T maps but absent from the G2019S one. The dashed arrows in (A) and (C) indicate that two different rotamers of H1998 can be accommodated by the maps of WT and I2020T. The rotamer pointing “up” (toward the kinase N-lobe) is shown in these panels. Note that only density accounting for the “down” rotamer is seen in G2019S (B). The G-loop and DYG motif are indicated in all three panels. (**D** to **F**) Inhibitor binding pocket. The semitransparent orange surface indicates the solvent-accessible surface for those residues in contact with MLi-2. For LRRK2^RCKW^ WT and I2020T, these surfaces were generated using the “up” rotamer of H1998. The “up” rotamer of H1998 leads to a closing of the binding pocket in WT and I2020T (D and F) that is absent in G2019S (E). (**G**) Superposition of the models for MLi-2 bound to LRRK2^RCKW^ WT, G2019S, and I2020T. G2019S is shown in darker shades. The gray gradient-colored arrow in the G-loop indicates the movement of the loop in G2019S relative to WT and I2020T. The two-headed orange arrow indicates that two rotamers of H1998 in LRRK2^RCKW^ WT and I2020T can account for the density in our cryo-EM maps. (**H**) Differential scanning fluorimetry data measured for LRRK2^RCKW^(WT) and LRRK2^RCKW^(G2019S) in the presence and absence of MLi-2. In the absence of an inhibitor, both proteins showed identical melting points suggesting that the mutant did not affect the stability of the recombinant protein. The binding of the inhibitor resulted in a lower Δ*T*_M_ for LRRK2^RCKW^(G2019S), suggesting a weaker binding affinity of MLi-2. AU, arbitrary units.

G2019S is the most frequently identified LRRK2 mutation in patients with PD (*28*). Although the G2019S mutation increases LRRK2 kinase activity, as do the other most common mutations (*5*–*8*), LRRK2(G2019S) is unusual: It is the only variant that does not increase microtubule association in cells (*31*, *32*), it shows the highest levels of autophosphorylation at S1292 (*33*), and this mutation has been reported both to provide resistance to inhibition by MLi-2 (*34*) and to be more sensitive to this inhibitor (*25*). We therefore aimed to visualize, structurally, any differences that may exist in how inhibitors interact with LRRK2 carrying different PD-linked mutations. For this, we solved cryo-EM structures of LRRK2^RCKW^ WT, and I2020T, bound to MLi-2 (Fig. 2, figs. S5 to S7, and table S1) and compared them to the structure of LRRK2^RCKW^(G2019S):MLi-2 presented above. We chose the I2020T mutation because of the different properties exhibited by G2019S and I2020T despite affecting neighboring residues. As was the case with the structure of G2019S bound to MLi-2, these maps were of high enough resolution to reveal the details of the kinase’s active site. The WT map had an overall resolution of 3.05 Å and local resolutions as high as 2.8 Å around the kinase’s active site (fig. S6 and table S1), while the I2020T map had an overall resolution of 2.74 Å and local resolutions as high as 2.5 Å around the kinase’s active site (fig. S7 and table S1).

Although the three structures—WT, G2019S, and I2020T—were very similar (Fig. 2), two features were unique to LRRK2^RCKW^(G2019S):MLi-2. First, the presence of a Ser instead of a Gly at position 2019 introduced a clash with the G-loop that pushed S1889 away from S2019 (Fig. 2, A to C and G). The second was an unexplained density we observed in the LRRK2^RCKW^(WT):MLi-2 and LRRK2^RCKW^(I2020T):MLi-2 cryo-EM maps, but not in that of LRRK2^RCKW^(G2019S):MLi-2 (Fig. 2, A to C). This additional density in WT and I2020T could accommodate an alternative rotamer for H1998 (Fig. 2, A, B, and G), which closes the binding site around MLi-2 (Fig. 2, D to F). Given the absence of density for this rotamer in G2019S, we predicted weaker binding of MLi-2 to LRRK2’s kinase carrying the G2019S mutation. Although the accurate determination of sub-nanomolar inhibitor affinities for complex proteins is challenging, our DSF assay showed that the addition of MLi-2 to LRRK2^KW^(G2019S) resulted in less thermal stabilization than that observed with LRRK2^KW^(WT) (Fig. 2H).

### Structures of PD variants of LRRK2^RCKW^ bound to the type II inhibitor GZD-824

**Fig. 3.**
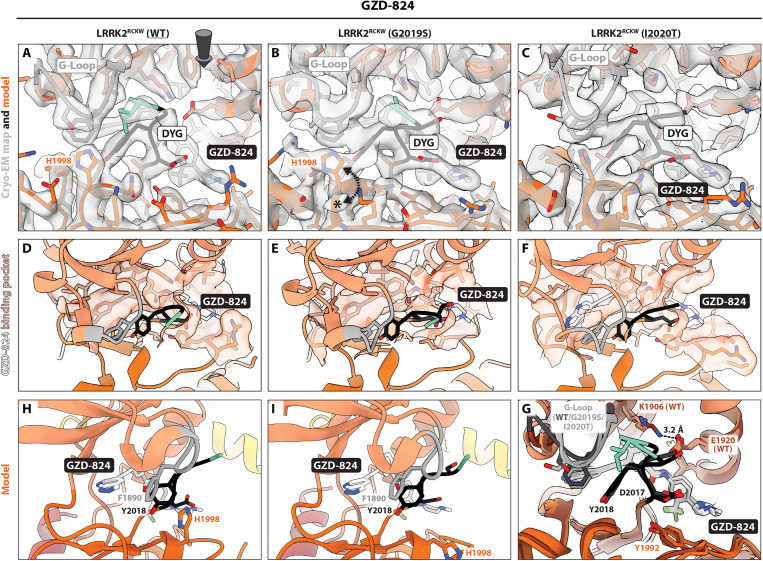
Binding of GZD-824 to LRRK2^RCKW^ WT, G2019S, and I2020T. (**A** and **C**) Cryo-EM maps and models for the GZD-824 binding site and surrounding regions for LRRK2^RCKW^(WT):GZD-824 (A), LRRK2^RCKW^(G2019S):GZD-824 (B), and LRRK2^RCKW^(I2020T):GZD-824 (C). The asterisk in (**B**) highlights a density present in G2019S but absent in WT. The dashed arrows in (B) indicate that two different rotamers of H1998 can be accommodated by the map of G2019S. The rotamer that points “up” (toward the N-lobe of the kinase) is shown in (B). Note that only density accounting for the “up” rotamer is seen in WT (A). The G-loop and DYG motif are indicated in the panels. The arrow in (A) indicates the viewing direction for (D) to (F). (**D** to **F**) Inhibitor binding pocket. The semitransparent orange surface indicates the solvent-accessible surface for those residues in contact with GZD-824. (**G**) Superposition of the models for GZD-824 bound to LRRK2^RCKW^ WT, G2019S, and I2020T. WT is shown in darker shades. (**H** and **I**) Pi-pi interactions in the GZD-824 binding pocket. The GZD-824 binding pocket is viewed from the direction of the kinase’s hinge motif for WT (H) and G2019S (I). The G-loop’s F1890 faces the pyrazolopyridine ring of the inhibitor and, in turn, interacts with the Y2018 of the DYG motif. H1998 is parallel to F1890 in the WT structure, where it adopts the “up” rotamer (H). G2019S can accommodate two rotamers for H1998 (B), and the model in (I) shows H1998 in its “down” rotamer, which places it away from the inhibitor’s binding site.

Next, we solved cryo-EM structures of LRRK2^RCKW^ WT and I2020T bound to the broad-spectrum type II inhibitor GZD-824 (Fig. 3, figs. S5, S8, and S9, and table S1) and compared them with the structure of G2019S bound to GZD-824 discussed above. As with the structure of G2019S bound to GZD-824, the resolution of the maps allowed us to visualize the details of the kinase’s active site. The WT map had an overall resolution of 2.9 Å and local resolutions as high as 2.8 Å around the kinase’s active site (fig. S8 and table S1), while the I2020T map had an overall resolution of 3.22 Å and local resolutions as high as 2.8 Å around the kinase’s active site (fig. S9 and table S1).

As with MLi-2, the three structures—WT, G2019S, and I2020T—were very similar (Fig. 3G). Although all three structures assumed a DYG “out”, αC “out” conformation with a broken R-spine and a disordered activation loop, the WT cryo-EM map showed a closer distance between K1906 and E1920 (Fig. 3, A and G). In all three cases, the G-loop was folded over the inhibitor to interact with its pyrazolopyridine and Y2018 from the DYG motif, as described above (Fig. 3, A to C and G).

We noticed differences in the rotamers adopted by H1998 in these structures, as had been the case with the MLi-2 ones. Here, however, the cryo-EM map for LRRK2^RCKW^(G2019S):GZD-824 could accommodate two rotamers for H1998 (Fig. 3B), while WT (Fig. 3A) showed density for a single rotamer. Although LRRK2^RCKW^(I2020T):GZD-824 appeared to also show a single rotamer (Fig. 3C), this cryo-EM map was of lower resolution and more anisotropic, making it difficult to establish this side chain conformation unambiguously. A potential role for H1998 in the GZD-824–bound structures is in the stabilization of F1890 in the G-loop, which is packed against the pyrazolopyridine ring of the inhibitor (Fig. 3H). The presence of an alternative rotamer in G2019S (Fig. 3I) could result in weaker binding of GZD-824 to this mutant. This prediction is consistent with a study showing that GZD-824 inhibits LRRK2(WT) in an in vitro phosphorylation assay with an IC_50_ ~4-fold lower than that for LRRK2(G2019S) (*25*).

### Structures of full-length LRRK2 bound to MLi-2 and GZD-824

**Fig. 4.**
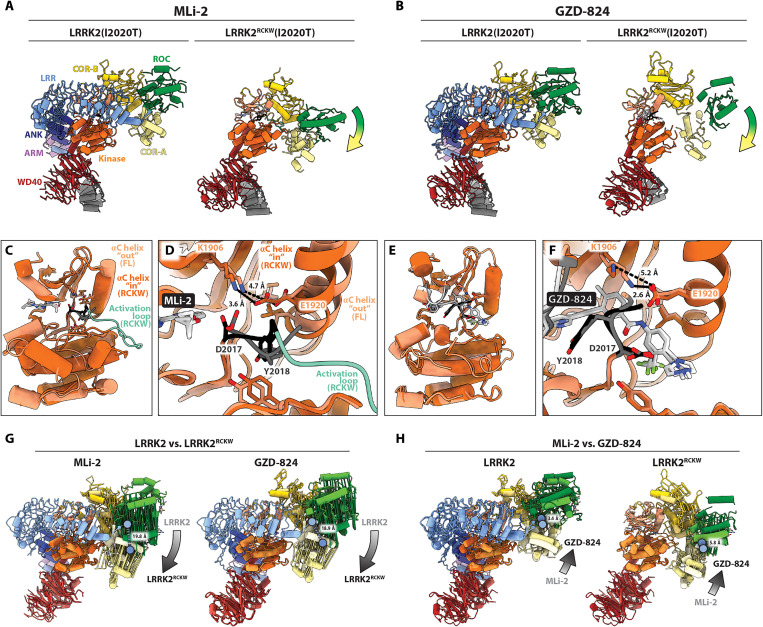
Structures of full-length LRRK2(I2020T) bound to MLi-2 or GZD-824. (**A**) Structures of LRRK2(I2020T) (left) and LRRK2^RCKW^(I2020T) (right) bound to MLi-2. [The structure of LRRK2^RCKW^(I2020T):MLi-2 is the same one shown in Fig. 2.] The colored arrow indicates the movement of the ROC-COR domains in LRRK2^RCKW^ relative to full-length LRRK2. (**B**) Same as in (A), for GZD-824. [The structure of LRRK2^RCKW^(I2020T):GZD-824 is the same one shown in Fig. 3.] (**C**) Overlay of the kinase domains from LRRK2(I2020T) and LRRK2^RCKW^(I2020T) bound to MLi-2, highlighting the “out” conformation of the αC helix. In (C) to (F), lighter shades represent LRRK2 and darker shades represent LRRK2^RCKW^. (**D**) Close-up of the kinase active sites in (C), with major features labeled. (**E**) Overlay of the kinase domains from LRRK2(I2020T) and LRRK2^RCKW^(I2020T) bound to GZD-824, highlighting their similarity. (**F**) Close-up of the kinase active sites in (C), with major features labeled. (**G**) Conformational differences between LRRK2 and LRRK2^RCKW^ bound to either MLi-2 (left) or GZD-824 (right). Structures were aligned using the C-lobes of their kinase domains. Colored vectors connect the same α carbons between the two structures. The gradient arrows highlight the direction of the overall relative movement of the ROC-COR moiety. The blue circles mark M1335 at the beginning of the ROC domain; the distance separating the α carbons of M1335 in the two structures being compared is indicated. (**H**) Differences in the conformations adopted by LRRK2 (left) or LRRK2^RCKW^ (right) in the presence of either MLi-2 or GZD-824. All labels and indicators are the same as those in (G).

On cryo-EM grids, LRRK2 monomers were shown to adopt an autoinhibited conformation where the N-terminal repeats (ARM-ANK-LRR) sterically block access to the kinase’s active site (*13*). The repeats, which connect to the ROC domain, are held in place by interactions between the ANK domain and the C-terminal helix of LRRK2 (*13*). This anchoring of the repeats restricts conformational changes within LRRK2. To determine whether the presence of the repeats would prevent some of the conformational changes we observed in the structures we obtained with LRRK2^RCKW^, we solved structures of full-length LRRK2(I2020T) bound to either MLi-2 or GZD-824 (Fig. 4, figs. S10 and S11, and table S1). As it was the case for all LRRK2^RCKW^ structures reported here, we solved the full-length ones in the presence of the E11 DARPin and GDP. The maps of full-length LRRK2 were of lower resolution than those for LRRK2^RCKW^ but still sufficient to determine the main features of the kinase’s active site. The map of full-length LRRK2 bound to MLi-2 had an overall resolution of 3.9 Å, with a slightly higher local resolution of 3.8 Å around the kinase’s active site (fig. S10 and table S1). The map of full-length LRRK2 bound to GZD-824 had an overall resolution of 3.4 Å, with a slightly higher local resolution of 3.3 Å around the kinase’s active site (fig. S11 and table S1).

As expected from the anchoring effect of the repeats, both LRRK2(I2020T):MLi-2 and LRRK2(I2020T):GZD-824 had their ROC-COR domains in a substantially more open (i.e., further away from the kinase) conformation than that seen in the equivalent structures with LRRK2^RCKW^ (Fig. 4, A and B). Next, we compared the kinase domains to see whether this difference in global conformation reflected differences in the states of the kinases themselves between LRRK2 and LRRK2^RCKW^ (Fig. 4, C to F). This was the case for MLi-2: While our structure of LRRK2^RCKW^(I2020T):MLi-2 showed an active-like, DYG “in”, αC “in” conformation with an ordered activation loop, the kinase in LRRK2(I2020T):MLi-2 had its αC in an “out” conformation and a disordered activation loop. This difference, in contrast, was absent from the GZD-824 structures; the kinases in LRRK2(I2020T):GZD-824 and LRRK2^RCKW^(I2020T):GZD-824 were almost identical, the only difference being the formation of the salt bridge between K1906 and E1920 in LRRK2(I2020T):GZD-824 (Fig. 4, E and F).

To better understand this difference between MLi-2 and GZD-824, we compared the four structures carrying the I2020T mutation—LRRK2(I2020T):MLi-2, LRRK2^RCKW^(I2020T):MLi-2, LRRK2(I2020T):GZD-824, and LRRK2^RCKW^(I2020T):GZD-824—by aligning them using the C-lobe of their kinases (Fig. 4, G and H). We used vectors connecting equivalent α carbons to illustrate the conformational changes, and we quantified differences between structures by measuring the distance between the α carbons of M1335, a residue located at the beginning of the ROC domain (Fig. 4, G and H). This analysis suggested that the repeats may be preventing the kinase in LRRK2(I2020T):MLi-2 from reaching its fully closed/active-like conformation. While both MLi-2 and GZD-824 led to relatively similar conformational differences between LRRK2 and LRRK2^RCKW^, MLi-2 did seem to result in a slightly more closed conformation in LRRK2^RCKW^ (0.9 Å between the α carbons of M1335) (Fig. 4G), although we cannot determine whether this difference is significant at this point. The differences between the two types of inhibitors increased when we compared LRRK2(I2020T):MLi-2 to LRRK2(I2020T):GZD-824, and LRRK2^RCKW^(I2020T):MLi-2 to LRRK2^RCKW^(I2020T):GZD-824 (Fig. 4H). As expected, the type I inhibitor MLi-2 led to a more closed kinase conformation in LRRK2 than the type II GZD-824 (Fig. 4H, left); the ROC domain in the MLi-2 structure is shifted toward the kinase by 3.4 Å as measured by the distance between the M1335 residues. This difference increased to 5.8 Å in LRRK2^RCKW^, where the N-terminal repeats are absent (Fig. 4H, right). Together, these observations suggested that the inability of MLi-2 to induce a fully closed/active-like state in the kinase of LRRK2(I2020T):MLi-2 was a result of the conformational constraining brought about by the anchored N-terminal repeats. Although we do not yet understand, mechanistically, what physiological processes lead to the release of the N-terminal repeats of LRRK2, and thus to full activation of the kinase, our work shows that MLi-2 was able to interact with the kinase both in the active-like state (LRRK2^RCKW^) and in an intermediate activation state (LRRK2) (Fig. 4, C and D).

## DISCUSSION

Here, we have presented cryo-EM structures of LRRK2 and LRRK2^RCKW^ bound to the LRRK2-specific type I inhibitor MLi-2 and the broad-spectrum type II inhibitor GZD-824. These structures revealed the active-like state of LRRK2’s kinase (in the case of MLi-2) and the structural differences involved in engaging the two main types of inhibitors.

By comparing LRRK2^RCKW^(WT) with variants carrying the PD-linked mutations G2019S and I2020T, both of which are found within the kinase catalytic domain, we have begun to understand how some of these mutants might affect inhibitor binding. Similarly, comparing structures of inhibitor-bound LRRK2 and LRRK2^RCKW^ provided insights into how the N-terminal repeats of LRRK2 affect the interactions with inhibitors by constraining its conformational flexibility. These points are discussed further below.

### The role of the G2019S mutation in LRRK2 inhibition

A comparison of our structures of LRRK2^RCKW^(WT, G2019S, and I2020T) bound to MLi-2 showed a difference in the conformations that H1998, located in the C-lobe, could adopt (Fig. 2). The cryo-EM maps of WT and I2020T showed density that could be accounted for by two different rotamers of H1998, while G2019S could only accommodate one. One of the rotamers in WT and I2020T, the one absent in G2019S, contributed to a larger MLi-2–binding interface that closed around the inhibitor. The absence of this additional interaction in G2019S suggested that MLi-2 might bind to G2019S with lower affinity, a prediction that was consistent with our DSF measurements of LRRK2^KW^ upon the addition of MLi-2 (fig. S1).

A paper from the West group reported that the G2019S mutation made LRRK2 resistant to inhibition by MLi-2 in vivo. Although this would appear to agree with our structural and functional data, this study relied on measuring the loss of LRRK2-S1035 phosphorylation as a proxy for inhibition (*34*). While this is a frequently used biomarker for LRRK2 inhibition, it is a correlative one that is not directly dependent on LRRK2’s kinase activity. A direct measurement of LRRK2’s kinase activity, by quantifying the levels of Rab10 phosphorylation, was not possible in that study (*34*). On the other hand, data published by the Alessi group using purified LRRK2 and an in vitro assay with a peptide kinase substrate came to the opposite conclusion that MLi-2 inhibited LRRK2(G2019S) ~2-fold better than LRRK2(WT) (*25*). As we discuss below, we hypothesize that these discrepancies may reflect the type of construct used (full-length LRRK2 in the Alessi study, LRRK2^KW^ in ours), and the potential roles played by the N-terminal repeats of LRRK2 in conformational changes and inhibitor binding. A better understanding of the potential effect of H1998 on the inhibition of different PD variants of LRRK2 will require combining in vitro assays with H1998 mutants in the context of an activated LRRK2, either by the removal of its N-terminal repeats or by more physiological means yet to be determined, with structural characterization of changes in the active site brought about by the combination of the PD and H1998 mutations.

We also saw differences in the rotamers that H1998 could adopt in our structures of LRRK2^RCKW^ bound to GZD-824 (Fig. 3). In this case, the cryo-EM map of LRRK2^RCKW^(G2019S):GZD-824 showed density indicative of two rotamers for H1998, only one of which would contribute to inhibitor binding, while we only saw the latter in the cryo-EM map of the WT complex. The prediction, based on this structural difference, that LRRK2(WT) might bind more tightly than G2019S to GZD-824 was consistent with in vitro measurements of the IC_50_ values for GZD-824 for these two variants (*25*). The G-loop adopted slightly different conformations in our LRRK2^RCKW^(WT):GZD-824 and LRRK2^RCKW^(G2019S):GZD-824 structures and this could, in principle, have an effect on H1998, which is close to the G-loop in its “up” rotamer (Fig. 3, A and H). However, our model of LRRK2^RCKW^(I2020T):GZD-824 was very similar to the one of G2019S (Fig. 3G), yet the corresponding cryo-EM map appeared to show density for a single H1998 rotamer, equivalent to that in WT. The caveat, which we noted earlier, is that the cryo-EM map for I2020T was of lower resolution and more anisotropic than those for WT and G2019S, so it is not possible at this point to rule out the possibility that the conformation of the G-loop was responsible for the difference between WT and G2019S mutant.

### The role of the N-terminal repeats in LRRK2 inhibition

The N-terminal repeats of LRRK2 appear to play a double role in autoinhibition. On the one hand, the LRR domain physically blocks access to the kinase’s active site (*13*). On the other, the N-terminal repeats restrict the conformational flexibility of the C-terminal half of LRRK2 (LRRK2^RCKW^), to which they are connected at the ROC domain, by interacting with the C-terminal helix (via the ANK domain) (*13*). Our analysis of the conformational differences between LRRK2 and LRRK2^RCKW^ bound to MLi-2 and GZD-824 (Fig. 4) suggested that the constraints imposed by the N-terminal repeats had a bigger effect on type I inhibitors. While our structures of LRRK2^RCKW^:MLi-2 (Figs. 1 and 2) showed the kinase in a fully closed/active-like state, as would be expected from a type I inhibitor, the kinase in LRRK2(I2020T):MLi-2 was only partially closed, with its αC “out” and a disordered activation loop. We attribute this intermediate state to the tug of war between the MLi-2–induced closing of the kinase, and the constraining effect of the N-terminal repeats. We did not see this difference between LRRK2(I2020T):GZD-824 and LRRK2^RCKW^(I2020T):GZD-824; the kinases in the two structures, in an inactive/open conformation, were almost identical (Fig. 3).

Together, these comparisons suggest that the N-terminal repeats of LRRK2 prevent the kinase from reaching the fully closed/active-like state. At the same time, our structures showed that MLi-2 can bind to LRRK2 regardless of whether the kinase is fully closed (LRRK2^RCKW^) or in an intermediate activation state (LRRK2). Our results raise the possibility that IC_50_’s measured using full-length LRRK2 (in vitro, in cells, or in vivo) may be a combination of IC_50_’s for at least two different populations: One population corresponds to the autoinhibited LRRK2, where the kinase cannot reach its fully active-like state, represented by our LRRK2(I2020T):MLi-2 structure, while the other population corresponds to activated LRRK2, where the repeats have undocked and the kinase can reach its active-like state, represented by our LRRK2^RCKW^(G2019S):MLi-2 structure.

Given the importance played by the N-terminal repeats in physiological LRRK2 activation, and likely in therapeutic LRRK2 inhibition, an important focus of future studies will be the role played by LRRK2’s GTPase (the ROC domain) in its regulation. Since the ROC domain is where the N-terminal repeats join the C-terminal half of LRRK2 (LRRK2^RCKW^), small conformational changes in the ROC domain could have an important effect on the position of the lever-like N-terminal repeats. A better understanding of this potential regulatory role for the ROC domain will require combining functional assays and structural studies where the nucleotide state of the GTPase can be precisely controlled.

Another element in LRRK2 likely to play a role in regulating the N-terminal repeats, and thus LRRK2’s activation, is a loop in the LRR domain containing three phosphorylation sites: S910, S935, and S955. Phosphorylation of S910 and S935 is involved in recruiting 14-3-3 proteins, which are involved in keeping LRRK2 in the soluble cytosolic pool (*35*, *36*). An important question is whether the phosphorylation of these sites is dependent on the conformation of the N-terminal repeats. Recent work has shown that while treatment with type I inhibitors leads to dephosphorylation of S935 in cells, treatment with type II inhibitors does not (*25*), suggesting that this effect may be dependent on the conformation of the N-terminal repeats. So far, the loop containing these residues has been disordered in both the previously published structures of full-length LRRK2 (*13*) and those reported here. Since all these structures have represented the autoinhibited form of LRRK2, it is not clear at this point whether the inhibitor type–specific responses reflect different accessibility to the phosphorylation sites in the active and inactive forms of LRRK2 or to changes in the conformation of the loop itself between the two forms of LRRK2.

The structures presented here provide a blueprint for medicinal chemistry efforts to design new LRRK2-specific inhibitors, in particular type II inhibitors for which we have no examples of LRRK2-selective compounds. As mentioned in the introduction, selective type II inhibitors are needed not only to expand the potential therapeutic arsenal to treat PD but also to create a toolkit that allows inhibition of LRRK2 in conformation-specific states to better understand the function of this protein in cells and in vivo.

## MATERIALS AND METHODS

### Cloning, plasmid construction, and mutagenesis

Briefly, the DNA coding for LRRK2-WT residues 1327 to 2527 (taken from Mammalian Gene Collection) was polymerase chain reaction (PCR)–amplified using the forward primer TACTTCCAATCCATGAAAAAGGCTGTGCCTTATAACCGA and the reverse primer TATCCACCTTTACTGTCACTCAACAGATGTTCGTCTCATTTTTTCA. The amplicon was then inserted into the expression vector pFB-6HZB (https://www.thesgc.org/reagents/vectors, RRID:Addgene_53641) by ligation-independent cloning. This plasmid was used as a template for G2019S and I2020T site-directed mutagenesis (Q5 Site-Directed Mutagenesis Kit, NEB). According to Bac-to-Bac expression system protocols (Invitrogen), these plasmids were used for the generation of recombinant baculoviruses. The protocol can also be found in protocols.io: dx.doi.org/10.17504/protocols.io.kxygx35ddg8j/v1.

### Inhibitors

Stocks of the kinase inhibitors MLi-2 (10 mM; Tocris) and GZD-824 (10 mM; Cayman Chemical) were stored in dimethyl sulfoxide at −20°C.

### Differential scanning fluorimetry (DSF) assay

The assay was performed according to a previously established protocol (*37*). Briefly, a solution of 4 μM LRRK2^KW^ protein in assay buffer [20 mM Hepes (pH 7.4), 150 mM NaCl, 0.5 mM Tris (2-carboxyethyl) phosphine (TCEP), 20 μM GDP, 2.5 mM MgCl_2_, and 0.5% glycerol] was mixed 1:1000 with SYPRO Orange (Sigma-Aldrich). The inhibitors to be tested were added to a final concentration of 10 μM. Twenty microliters of each sample were placed in a 96-well plate and heated gradually from 25° to 95°C. Fluorescence was monitored using an MX3005P real-time PCR instrument (Stratagene) with excitation and emission filters set to 465 and 590 nm, respectively. Data were analyzed with the MxPro software. The protocol can also be found in protocols.io: dx.doi.org/10.17504/protocols.io.kxygx3y6kg8j/v1.

### MS-based activity assay

To determine the IC_50_ values for a set of LRRK2 inhibitors, the phosphorylation of the LRRK2 substrate Rab8A was assessed by applying an MS-based activity assay. LRRK2^RCKW^ protein (50 nM) was mixed with 5 μM substrate and varying concentrations of the inhibitors in assay buffer [20 mM Hepes (pH 7.4), 150 mM NaCl, 0.5 mM TCEP, 20 μM GDP, 2.5 mM MgCl_2_, and 0.5% glycerol]. The reaction was started by adding 1 mM ATP and incubated at room temperature (RT). The reaction was stopped by adding MS buffer (0.1% formic acid in water) and subjected to an Agilent 6230 electrospray ionization time-of-flight mass spectrometer coupled with a liquid chromatography unit 1260 Infinity for analysis. Five microliters of the reaction mix was injected onto a C3 column and eluted at 0.4 ml/min flow rate using a solvent gradient of water to acetonitrile with 0.1% formic acid. Data were acquired using the MassHunter LC/MS Data Acquisition software and analyzed using the BioConfirm vB.08.00 tool (both Agilent Technology) (https://www.agilent.com/en/product/software-informatics/mass-spectrometry-software, RRID: SCR_016657). The peak intensities of the unphosphorylated and phosphorylated Rab8A species were quantified and the relative kinase activity was calculated. To determine the IC_50_ values, a nonlinear regression with variable slope was fitted to the data points with GraphPad Prism. The protocol can also be found in protocols.io: dx.doi.org/10.17504/protocols.io.6qpvr385ovmk/v2.

### Protein purification: LRRK2^RCKW^ and LRRK2

For both LRRK2^RCKW^ and LRRK2 purifications, pelleted Sf9 cells were washed with phosphate-buffered saline, resuspended in lysis buffer [50 mM Hepes (pH 7.4), 500 mM NaCl, 20 mM imidazole, 0.5 mM TCEP, 5% glycerol, 5 mM MgCl_2_, and 20 μM GDP] and lysed by homogenization. The supernatant was cleared by centrifugation and loaded onto a Ni-NTA (Qiagen) column. After rinsing with lysis buffer, the His_6_-Z–tagged protein was eluted in lysis buffer containing 300 mM imidazole. The eluate was then diluted to 250 mM NaCl mM with dilution buffer [50 mM Hepes (pH 7.4), 0.5 mM TCEP, 5% glycerol, 5 mM MgCl_2_, and 20 μM GDP] and loaded onto an SP Sepharose column. His_6_-Z-TEV-LRRK2^RCKW^/His_6_-Z-TEV-LRRK2 were eluted with a gradient of 250 mM to 2.5 M NaCl in dilution buffer and then treated with Tobacco Etch Virus (TEV) protease overnight to cleave the His_6_-Z. Contaminating proteins, the cleaved tag, uncleaved protein, and TEV protease were removed by another combined SP sepharose Ni-NTA step. Last, LRRK2^RCKW^ was concentrated and subjected to gel filtration in 20 mM Hepes (pH 7.4), 700 mM NaCl, 0.5 mM TCEP, 5% glycerol, 2.5 mM MgCl_2_, and 20 μM GDP, while LRRK2 was concentrated and subjected to gel filtration in 20 mM Hepes (pH 7.4), 200 mM NaCl, 0.5 mM TCEP, 5% glycerol, 2.5 mM MgCl_2_, and 20 μM GDP using an ÄKTAxpress system with an S200 gel filtration column. Final yields, as calculated from ultraviolet absorbance, are typically 1.2 to 3.3 mg of LRRK2^RCKW^ and 0.9 to 2.2 mg of LRRK2 per liter of insect cell medium.

### Cryo-EM sample preparation for LRRK2^RCKW^:MLi-2/GZD-824:E11 DARPin complexes

Purified LRRK2^RCKW^ (WT, G2019S, or I2020T) was exchanged into 20 mM Hepes (pH 7.4), 150 mM NaCl, 0.5 mM TCEP, 5% glycerol, 2.5 mM MgCl_2_, and 20 μM GDP. LRRK2^RCKW^ was incubated with E11-DARPin in a 1:1.25 molar ratio and either MLi-2 (20 μM) or GZD-824 (40 μM) for 10 min at RT and 15 min at 4°C. Complexes were diluted to a final concentration range of 4 to 6 μM in the same buffer before plunge freezing. Three or 3.5 μl of LRRK2^RCKW^:inhibitor:E11 DARPin complex was applied to a glow-discharged UltrAuFoil Holey Gold 200 mesh R2/2 grid (Quantifoil) and incubated in a FEI Vitrobot IV chamber at 4°C and 95% humidity for 20 s. The excess liquid was blotted for 4 s using filter paper 595 at blot force 3, and vitrified by plunging into liquid ethane cooled down to liquid nitrogen temperature.

### Cryo-EM sample preparation for full-length LRRK2(I2020T):MLi-2/GZD-824-E11 DARPin complex

Purified full-length LRRK2(I2020T) was incubated with E11 DARPin at a molar ratio of 1:1.25 and either MLi-2 (20 μM) or GZD-824 (40 μM) for 10 min at RT and 5 to 10 min at 4°C. Complexes were diluted to a 5 μM final concentration in the same buffer [20 mM Hepes (pH 7.4), 150 mM NaCl, 0.5 mM TCEP, 5% glycerol, and 2.5 mM MgCl_2_] with the addition of a final 20 μM GDP for LRRK2(I2020T):MLi-2:E11 or 100 μM GMP-PNP for LRRK2(I2020T):GZD-824:E11. Three to 3.5 μl of the complex was applied to a glow-discharged UltrAuFoil Holey Gold 200 mesh R2/2 grid (Quantifoil) and incubated in a FEI Vitrobot IV chamber at 4°C and 95% humidity for 20 s. The excess liquid was blotted for 4 s using filter paper 595 at blot force 4, vitrified by plunging into liquid ethane cooled down to liquid nitrogen temperature. The protocol can also be found in protocols.io: dx.doi.org/10.17504/protocols.io.yxmvm35n9l3p/v1.

### Cryo-EM data collection

Cryo-EM data for LRRK2^RCKW^(G2019S and I2020T):MLi-2:E11 DARPin, LRRK2^RCKW^(WT, G2019S and I2020T):GZD-824:E11 DARPin, and LRRK2(I2020T):MLi-2:E11 DARPin were collected on a Titan Krios G3 (Thermo Fisher Scientific) operated at 300 keV, equipped with a Falcon 4 direct electron detector (Thermo Fisher Scientific) and a Gatan BioContinuum energy filter. Images were collected at a nominal magnification of 130,000× in EF-TEM mode (0.935-Å calibrated pixel size) using a 20-eV slit width in the energy filter and a cumulative electron exposure of ~55 electrons/Å^2^. Data for LRRK2(I2020T)-E11:GZD-824 were collected on a Talos Arctica (FEI) operated at 200 keV, equipped with a Falcon 4i (Thermo Fisher Scientific) with a cumulative electron exposure of ~55 electrons/Å^2^. Data were collected automatically using EPU software (Thermo Fisher Scientific). Data for LRRK2^RCKW^:MLi-2:E11 DARPin were collected on a Titan Krios G3 (Thermo Fisher Scientific) operated at 300 keV, equipped with a K3 Summit Direct Electron Detector (Gatan) and a Gatan BioContinuum energy filter. Images were collected at a nominal magnification of 105,000× in EF-TEM mode (0.822-Å calibrated pixel size) using a 20-eV slit width in the energy filter and a cumulative electron exposure of ~57 electrons/Å^2^. The protocol can also be found in protocols.io: dx.doi.org/10.17504/protocols.io.14egn3m5ql5d/v1.

### Data processing for LRRK2^RCKW^:MLi-2:E11 DARPin complexes

Movies (11,181, 8,402, and 10,488) were collected for LRRK2^RCKW^-WT:MLi-2:E11 DARPin, LRRK2^RCKW^(G2019S):MLi-2:E11 DARPin, and LRRK2^RCKW^(I2020T):MLi-2:E11 DARPin complexes, respectively. They were aligned using MotionCor2 dose-weighted alignment option and contrast transfer function (CTF) parameters were estimated on dose-weighted images using CTFFIND4 (*38*). Micrographs with a CTF fit worse than 3.5 to 4 Å (as determined by CTFFIND4, and varying among datasets) were discarded from further processing. Particles were picked using a Topaz model previously trained for each dataset. Several rounds of reference-free two-dimensional (2D) classification yielded a stack of 574,664 (WT), 578,873 (G2019S), and 694,336 particles (I2020T), each containing both monomers and tetramers. All particles were extracted with a 400-pixel box using CryoSPARC (*39*) (https://cryosparc.com/, RRID:SCR_016501) and were separated on the basis of the oligomerization state (figs. S3, S6, and S7). Subsequently, ab initio jobs were run to further remove bad particles. The best ab initio volume for the tetramer was used as an input for non-uniform refinement (NU refinement) (D2 symmetry). Then, particles were expanded on the basis of the volume symmetry and used in subsequent jobs. After 3D variability and C1 symmetry local refinement, we obtained maps at 3.05, 2.74, and 2.8 Å, respectively. The best ab initio map for the monomer, along with monomer particles (fig. S3, S6, and S7), was used as an input for an NU refinement. In all maps, the Fourier Shell Correlation (FSC) and local resolution estimations were performed using the routines implemented in CryoSPARC (*39*).

### Data processing for LRRK2(I2020T):MLi-2:E11 DARPin complex

A total of 10,407 movies were collected for LRRK2(I2020T):MLi-2:E11 DARPin and preprocessed as previously described. Micrographs with a CTF fit worse than 4 Å (as determined by CTFFIND4) were discarded from further processing. Particle picking was done following the same protocol for the LRRK2^RCKW^ datasets. Several rounds of 2D classification yielded 162,409 particles. After an ab initio job and an NU refinement, a final map at a resolution of 3.9 Å was obtained (fig. S10).

### Data processing for LRRK2^RCKW^:GZD-824:E11 DARPin complexes

Movies (5565, 7988, and 8386) were collected for LRRK2^RCKW^WT:GZD-824:E11 DARPin, LRRK2^RCKW^(G2019S):GZD-824:E11 DARPin, and LRRK2^RCKW^(I2020T):GZD-824:E11 DARPin complexes, respectively. They were aligned using MotionCor2 dose-weighted alignment option and CTF parameters were estimated on dose-weighted images using CTFFIND4 (*38*). Micrographs with a CTF fit worse than 3.5 to 4.5 Å (as determined by CTFFIND4, and varying among datasets) were discarded from further processing. Particles were picked using a Topaz model previously trained for each dataset. Several rounds of reference-free 2D classification yielded a stack of 249,808, 759,293, and 735,633 monomer particles respectively; in addition, LRRK2^RCKW^WT:GZD-824:E11 dataset also yielded 58,678 trimer particles. Particles were extracted with a 320-pixel box for monomers, and a 400-pixel box for trimers using CryoSPARC (figs. S4, S8, and S9) (*39*). For all LRRK2^RCKW^ monomer particles, ab initio jobs followed by heterogeneous refinement were run to sort particles. The best-refined class was subject to an NU refinement (C1 symmetry), followed by a local refinement with a mask surrounding the kinase-WD40-DARPin, yielding maps at resolutions of 3.10, 2.99, and 3.06 Å, respectively. To account for the heterogeneity of the ROC-COR domain, a 3D variability job was run to further split the particles, followed by a local refinement with a mask surrounding the ROC-COR domain; this yielded maps at resolutions of 3.80, 3.50, and 3.63 Å, respectively. For LRRK2^RCKW^ (trimer particles), an ab initio job was run followed by an NU refinement (C3 symmetry). Particles were expanded on the basis of the volume symmetry and used in a C1 symmetry local refinement, yielding a map at 2.90-Å resolution.

### Data processing for LRRK2(I2020T):GZD-824:E11 DARPin complexes

A total of 4102 movies were collected for LRRK2(I2020T):GZD-824:E11 and preprocessed as previously described. Micrographs with a CTF fit worse than 5.3 Å (as determined by CTFFIND4) were discarded from further processing. Particle picking was done in the same manner as for the LRRK2^RCKW^ datasets, followed by several rounds of 2D classification, resulting in 1,029,496 particles. Particles were moved to RELION using pyem/csparc2star (https://zenodo.org/records/3576630) and extracted to 3.8 Å/pixel. Particles were subject to 3D classification (five classes) with alignment, 224,119 particles yielded the best 3D class of monomeric LRRK2(I2020T). Consensus 3D refinement using the best 3D class was done, followed by two rounds of 3D classification without alignment resulting in the best 3D class containing 60,465 particles. Particles were moved back to CryoSPARC (*34*), followed by an NU refinement, yielding a map at 3.40-Å resolution (fig. S11).

### Model building and refinement of LRRK2^RCKW^:MLi-2/GZD-824:E11 DARPin and LRRK2(I2020T):MLi-2/GZD-824:E11 DARPin

LRRK2^RCKW^ and LRRK2 models were built using the highest-resolution maps obtained for each complex. These maps corresponded to tetramers for LRRK2^RCKW^(WT):MLi-2:E11, LRRK2^RCKW^(G2019S):MLi-2:E11, and LRRK2^RCKW^(I2020T):MLi-2:E11, a trimer for LRRK2^RCKW^(WT):GZD-824:E11 and a monomer for FL(I2020T):MLi-2/GZD-824:E11 DARPin (see figs. S2, S4, and S6 to S11 for details on the cryo-EM workflow for each specific sample). To rule out structural differences in the kinase induced by oligomerization, we tested the fit of the models built using the higher-resolution oligomers into cryo-EM maps of the monomeric form of the same construct (fig. S5).

Available Protein Data Bank (PDB) 6VP7, 7LHW, and A1N for LRRK2^RCKW^, LRRK2, and MLi-2, respectively, were used as starting points. Protein models were split into domains, docked into the corresponding cryo-EM maps, and merged. To obtain a GZD-824 model, electronic Ligand Building and Optimization Workbench (elBOW) software available in Phenix (*40*) was run using simplified molecular-input line-entry system notation of molecule as an input. Inhibitor models (MLi-2 or GZD-824) were fitted in the map density and incorporated into the model. A combination of manual inspection of amino acids in Coot (*41*, *42*) and refinement of the model into their maps in Phenix (*40*) was used to generate the final models. The protocol can also be found in protocols.io: dx.doi.org/10.17504/protocols.io.81wgbx5m1lpk/v1.

### Analysis of conformational changes in LRRK2^RCKW^ and LRRK2

The segments linking equivalent α carbons in LRRK2^RCKW^ and LRRK2 were generated by aligning models by their C-lobes (residues 1949 to 2139) using UCSF ChimeraX (*43*). Amino acids not present in the two models being compared were removed and primary sequences were aligned using ClustalW (EMBL-EBI) (https://www.ebi.ac.uk/Tools/msa/clustalo/, RRID:SCR_017277) (*44*). Aligned sequences were used as input for the public script PDBarrows.ipynb (https://github.com/sami-chaaban/PDBArrows) on Jupyter Notebook, generating output arrows, which were colored to match the domains being linked. (Fig. 4, G and H).

### Structure depictions

All structure-related figures were prepared using ChimeraX (*43*).
