# Supplementary material for: Inhibition of Parkinson’s disease–related LRRK2 by type I and type II kinase inhibitors: Activity and structures
Source: Sci Adv. 2023 Dec 1;9(48):eadk6191. doi: 10.1126/sciadv.adk6191 (PMC10691770; doi:10.1126/sciadv.adk6191)
Supplement: Supplementary file 1 — Figs. S1 to S11 Legend for movie S1 Table S1 [file sciadv.adk6191_sm.pdf]

Science Advances
AAAS

Supplementary Materials for

Inhibition of Parkinson's disease–related LRRK2 by type I and type II kinase inhibitors: Activity and structures

Marta Sanz Murillo *et al.*

Corresponding author: Andres E Leschziner, aleschziner@ucsd.edu

Sci. Adv. **9**, eadk6191 (2023)
DOI: 10.1126/sciadv.adk6191

This PDF file includes:

Figs. S1 to S11
Legend for movie S1
Table S1

Other Supplementary Material for this manuscript includes the following:

Movie S1

Supplementary Figures

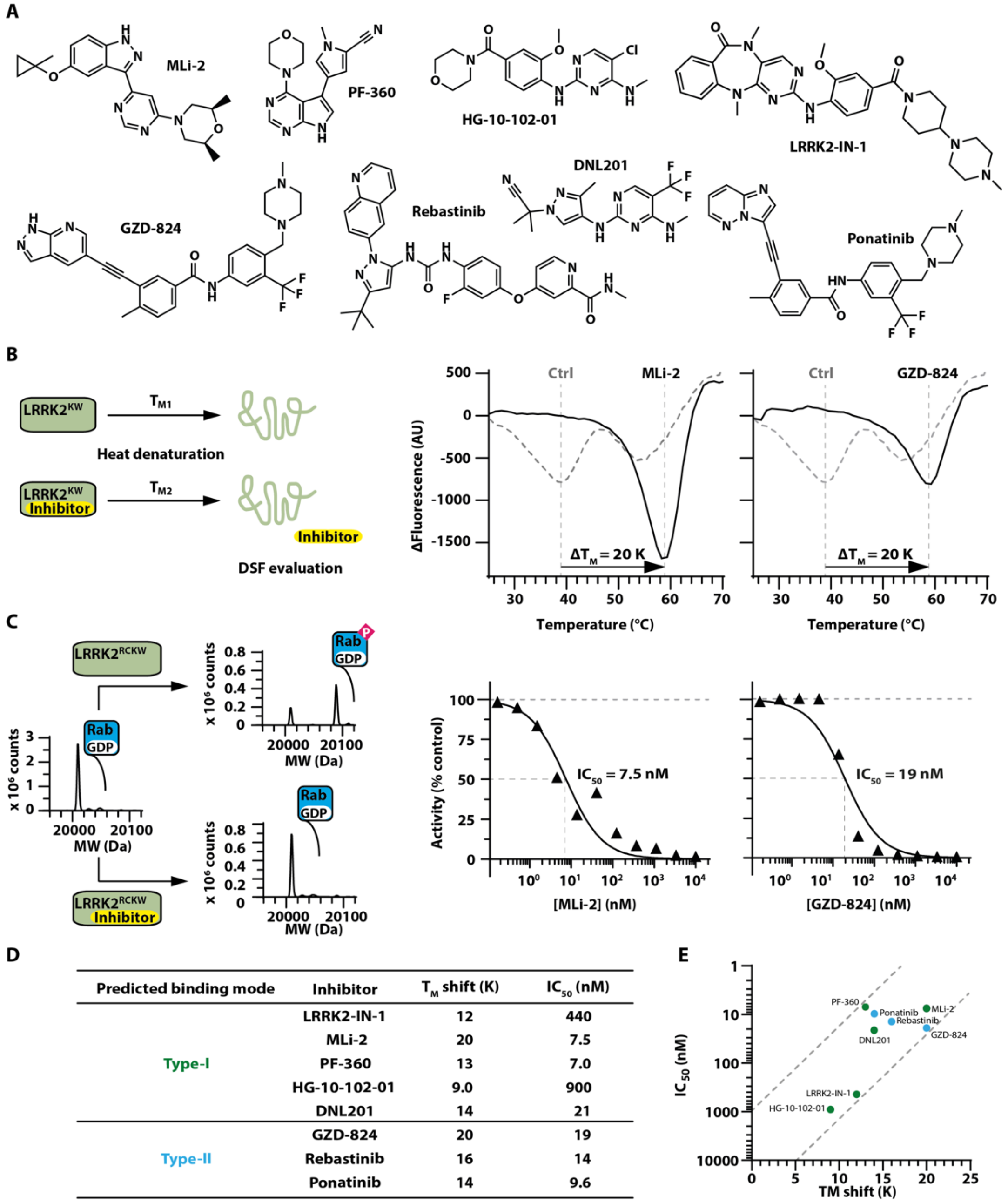

Predicted binding mode	Inhibitor	T_M shift (K)	IC_{50} (nM)
Type-I	LRRK2-IN-1	12	440
	MLi-2	20	7.5
	PF-360	13	7.0
	HG-10-102-01	9.0	900
	DNL201	14	21
Type-II	GZD-824	20	19
	Rebastinib	16	14
	Ponatinib	14	9.6

Figure S1. Stabilization and inhibition of LRRK2's kinase by type-I and type-II inhibitors

(A) Prominent inhibitors of the LRRK2 kinase. **(B)** The binding of kinase inhibitors stabilized $LRRK2^{KW}$ as determined in a DSF assay. Shown are the first derivatives of the melting curves. The biphasic melting of $LRRK2^{KW}$ indicated that the kinase and WD40 domains melt independently of each other. **(C)** The impact of type-I and type-II inhibitors on $LRRK2^{RCKW}$ kinase activity was assessed with an MS-based activity assay. The inhibitors prevented $LRRK2^{RCKW}$ from phosphorylating Rab8A with nanomolar IC_{50} values. **(D)** T_M shifts (for $LRRK2^{KW}$) and IC_{50}s (for $LRRK2^{RCKW}$) obtained for the type-I and type-II inhibitors shown in (A). **(E)** Plot of IC_{50}s as a function of T_M shifts for the inhibitors in (E). The Pearson correlation coefficient between the two measurements is -0.67.

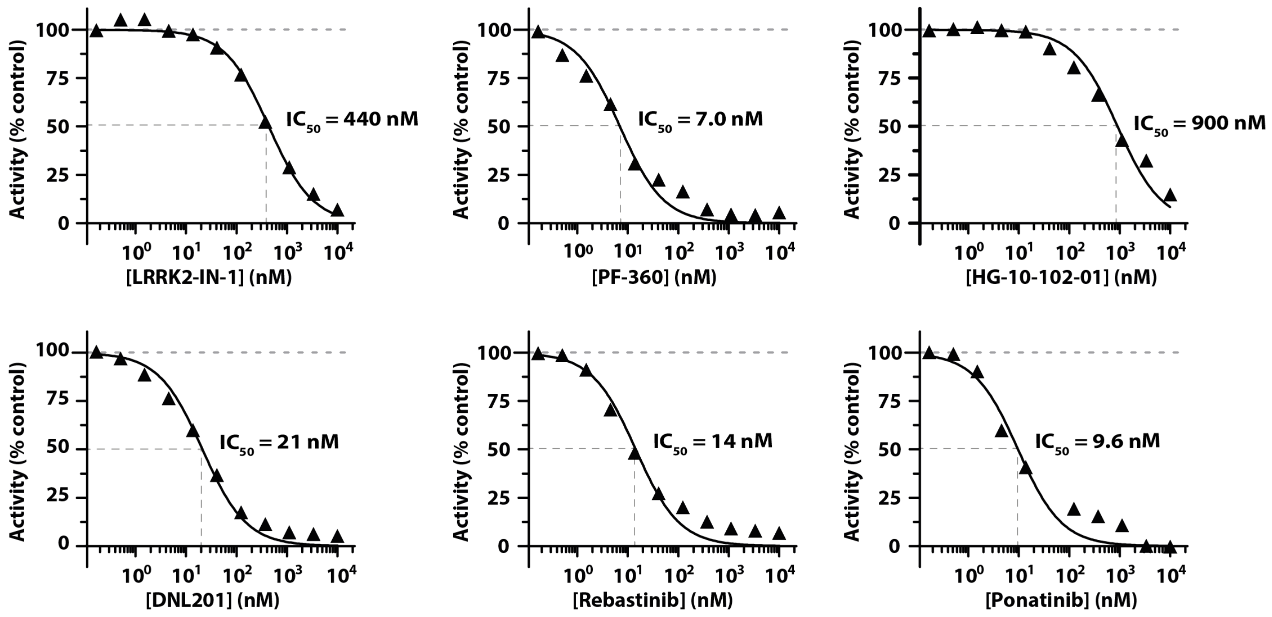

Figure S2. LRRK2 inhibition curves obtained from the MS-based activity assay

The physiological LRRK2 substrate Rab8A was subjected to phosphorylation by $LRRK2^{RCKW}$. Varying concentrations of the indicated inhibitors were present in the reaction mixtures allowing for the determination of IC_{50} values.

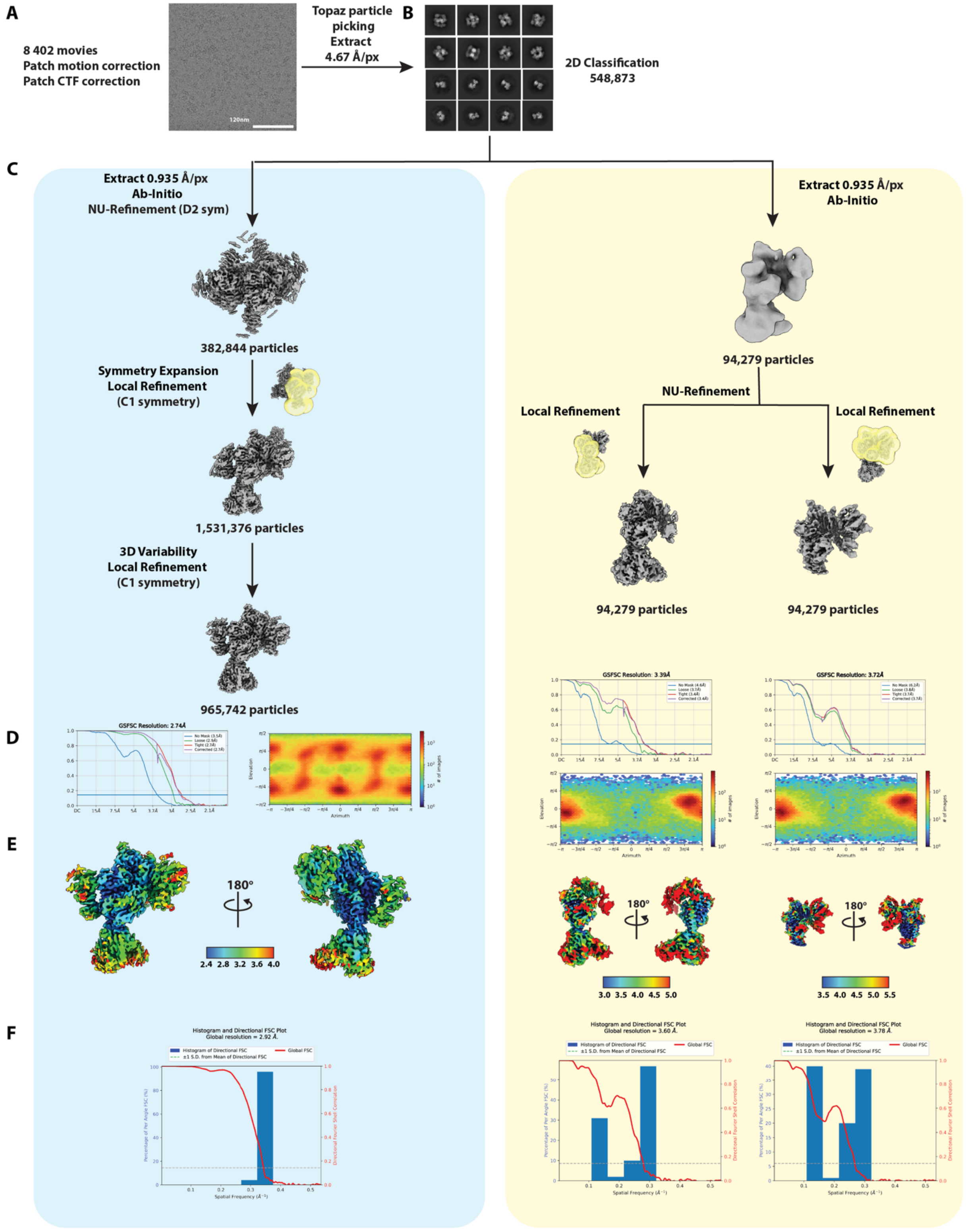

Figure S3. Cryo-EM workflow for LRRK2^{RCKW}(G2019S):MLi-2

Representative micrograph **(A),** 2D class averages **(B)**, data processing strategy **(C)**, FSC plots and Euler angle distributions **(D),** local resolution maps **(E)**, and 3D FSC plot **(F)** for LRRK2^{RCKW}(G2019S):MLi-2 tetramer (blue panel) and monomer (yellow panel).

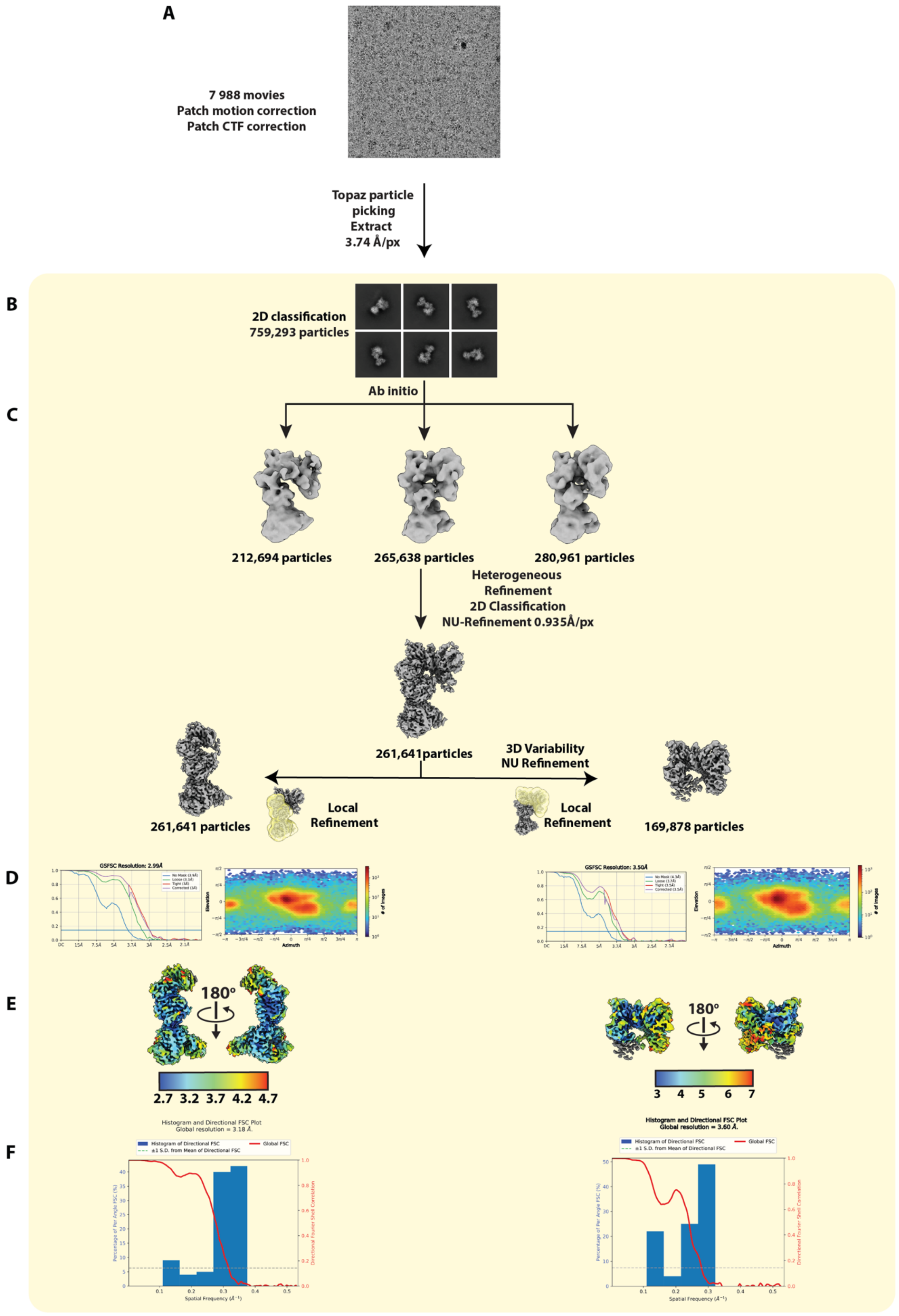

Figure S4. Cryo-EM workflow for LRRK2^{RCKW}(G2019S):GZD-824

Representative micrograph **(A)**, 2D class averages **(B),** data processing strategy **(C)**, FSC plots and Euler angle distributions **(D),** local resolution maps **(E)**, and 3D FSC plot **(F)** for LRRK2^{RCKW}(G2019S):GZD-824 monomer.

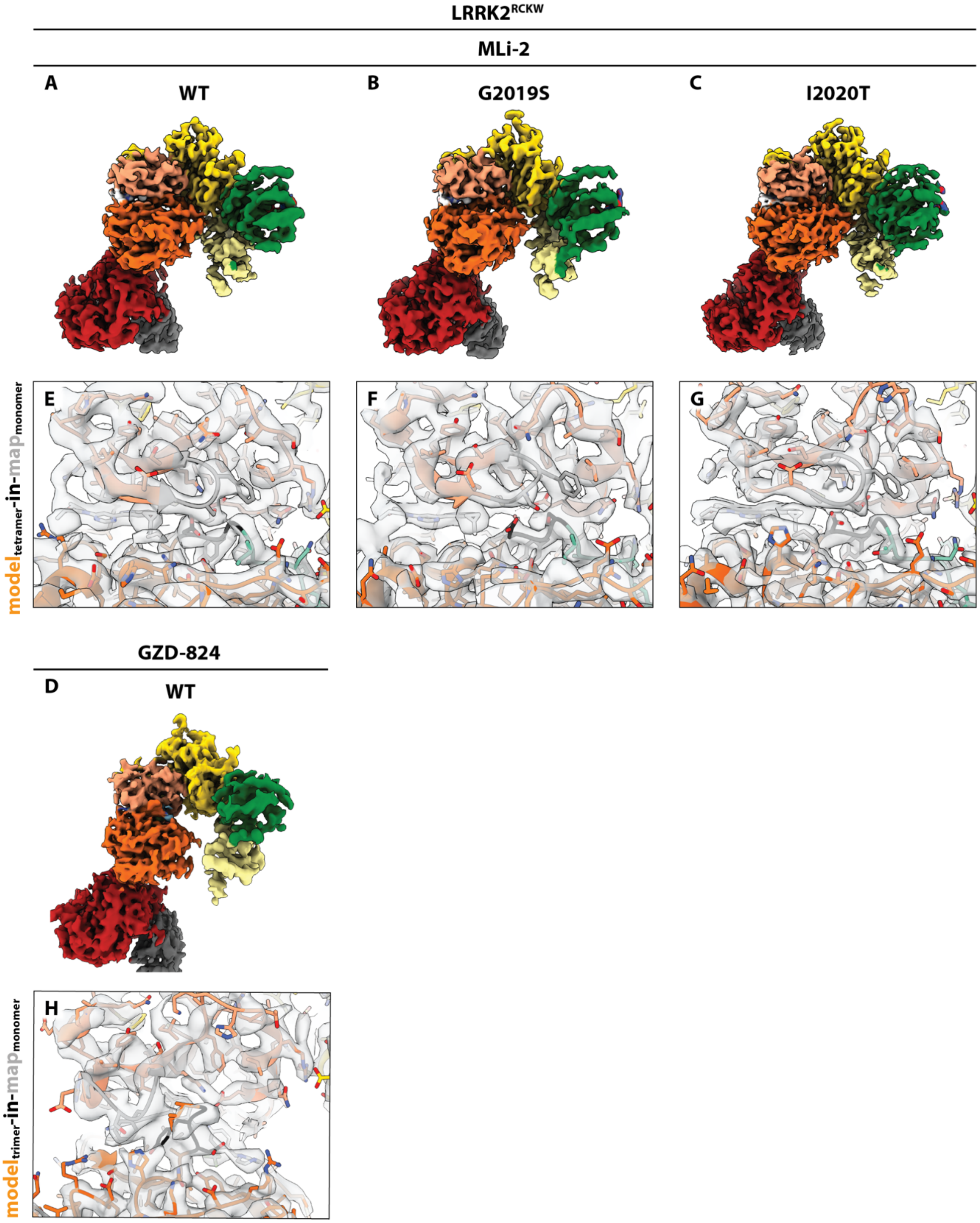

Figure S5. Cryo-EM maps of monomeric LRRK2^{RCKW}'s account for models built into higher-resolution trimeric and tetrameric maps

(A-D) Cryo-EM maps of the monomeric form of LRRK2^{RCKW}(WT):MLi-2 (A), LRRK2^{RCKW}(G2019S):MLi-2 (B), LRRK2^{RCKW}(I2020T):MLi-2 (C), and LRRK2^{RCKW}(WT):GZD-824 (D). Maps are colored according to the domain color scheme shown in **Figure S1A**. The DARPin E11 is shown in dark grey. **(E-H)** The models built using the higher-resolution tetrameric (E-G) and trimeric (H) cryo-EM maps are fitted into the monomeric maps (see Methods for details on the cryo-EM data processing) to highlight that the kinase features discussed in the text (G-loop, DYG motif, αC helix, K1906-E1920 pair, and activation loop) are not dependent on the oligomeric state of LRRK2^{RCKW}. The panels show the fit in and around the kinase's active site.

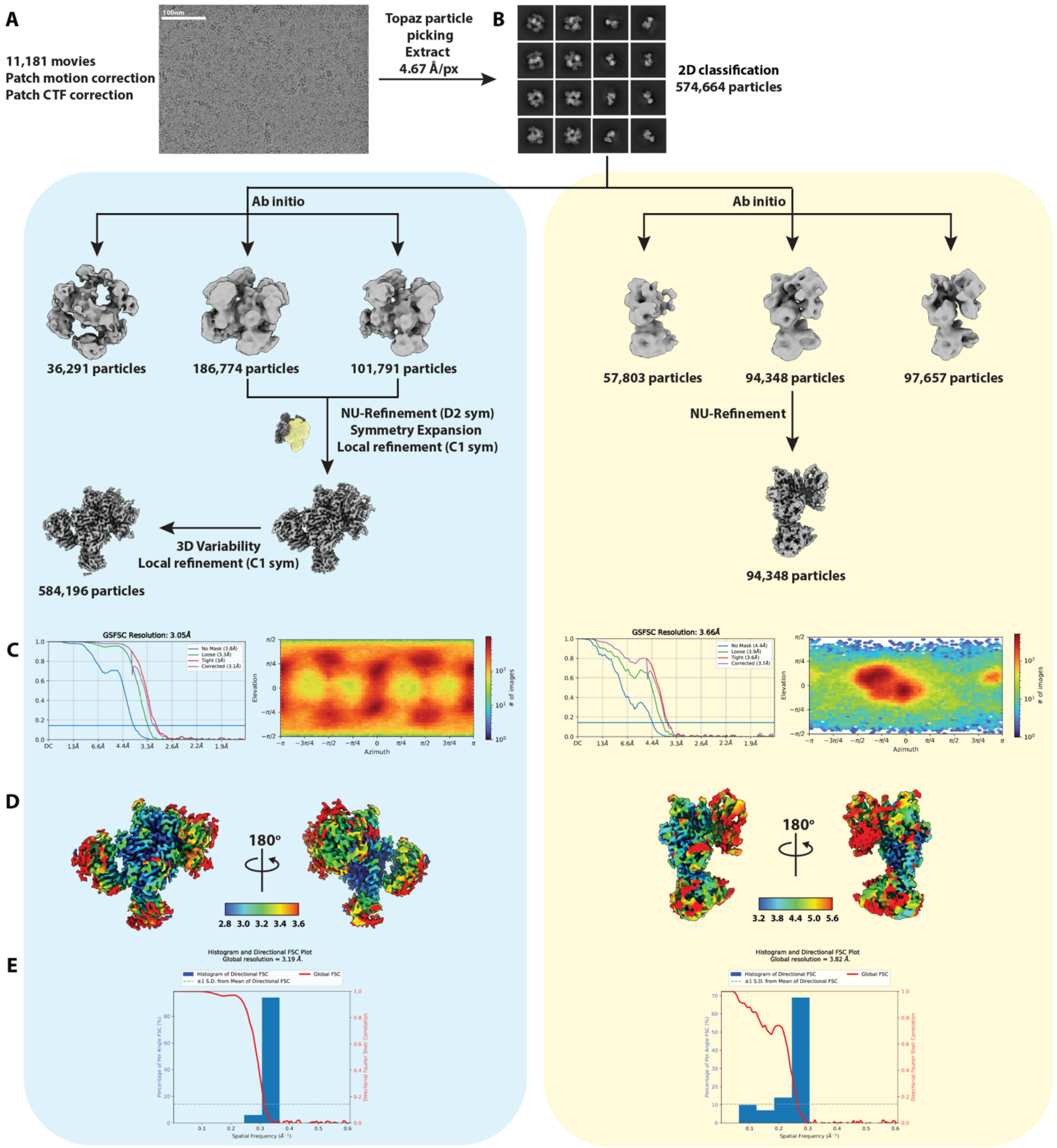

Figure S6. Cryo-EM workflow for LRRK2^{RCKW}(WT):MLi-2

Representative micrograph **(A),** 2D class averages and data processing strategy **(B)**, FSC plots and Euler angle distributions **(C),** local resolution maps **(D)**, and 3D FSC plots **(E)** for LRRK2^{RCKW}(WT):MLi-2 tetramer (blue panel) and monomer (yellow panel).

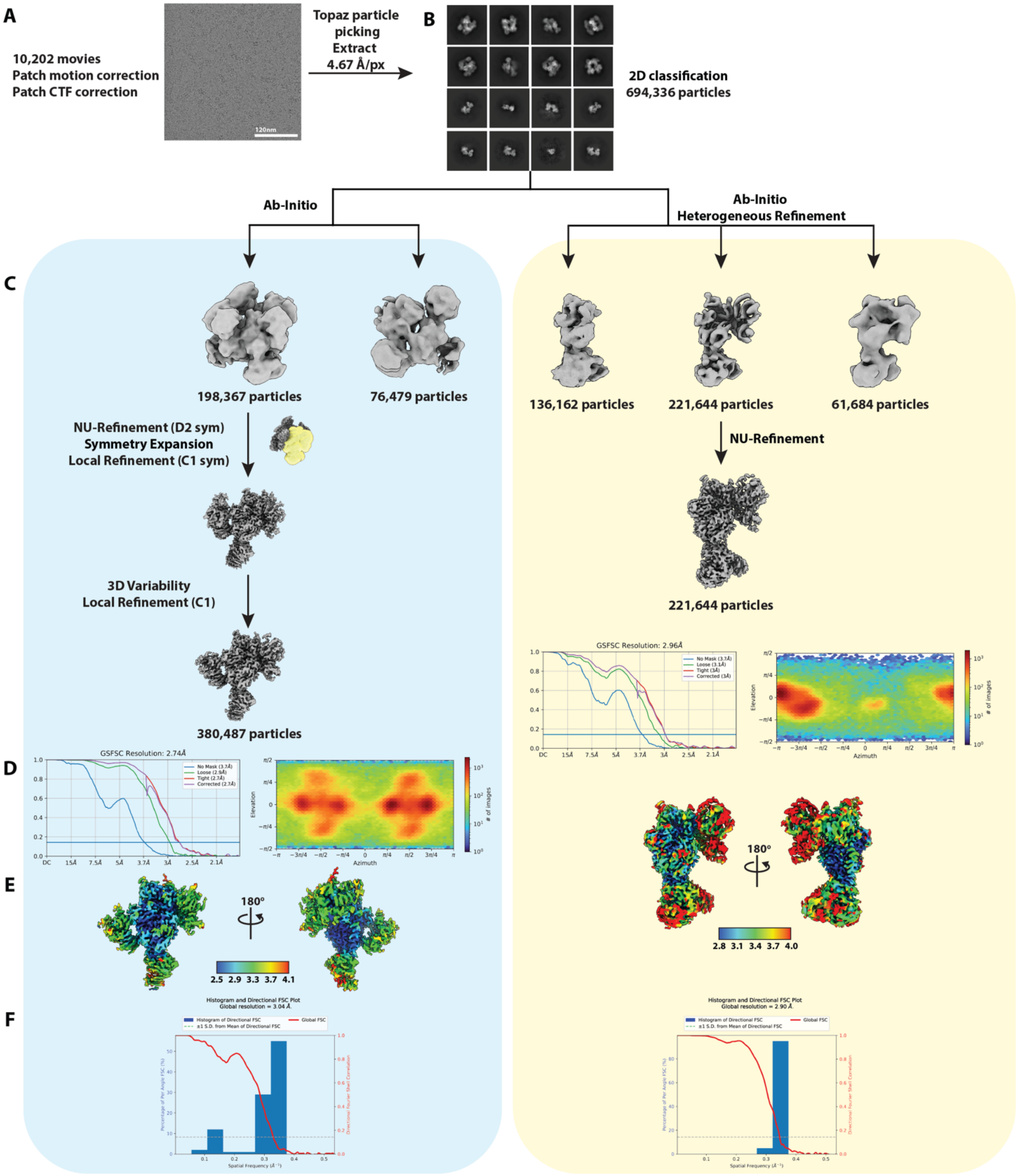

Figure S7. Cryo-EM workflow for LRRK2^{RCKW}(I2020T):MLi-2

Representative micrograph **(A),** 2D class averages **(B)**, data processing strategy **(C)**, FSC plots and Euler angle distributions **(D),** local resolution maps **(E)**, and 3D FSC plot **(F)** for LRRK2^{RCKW}(I2020T):MLi-2 tetramer (blue panel) and monomer (yellow panel).

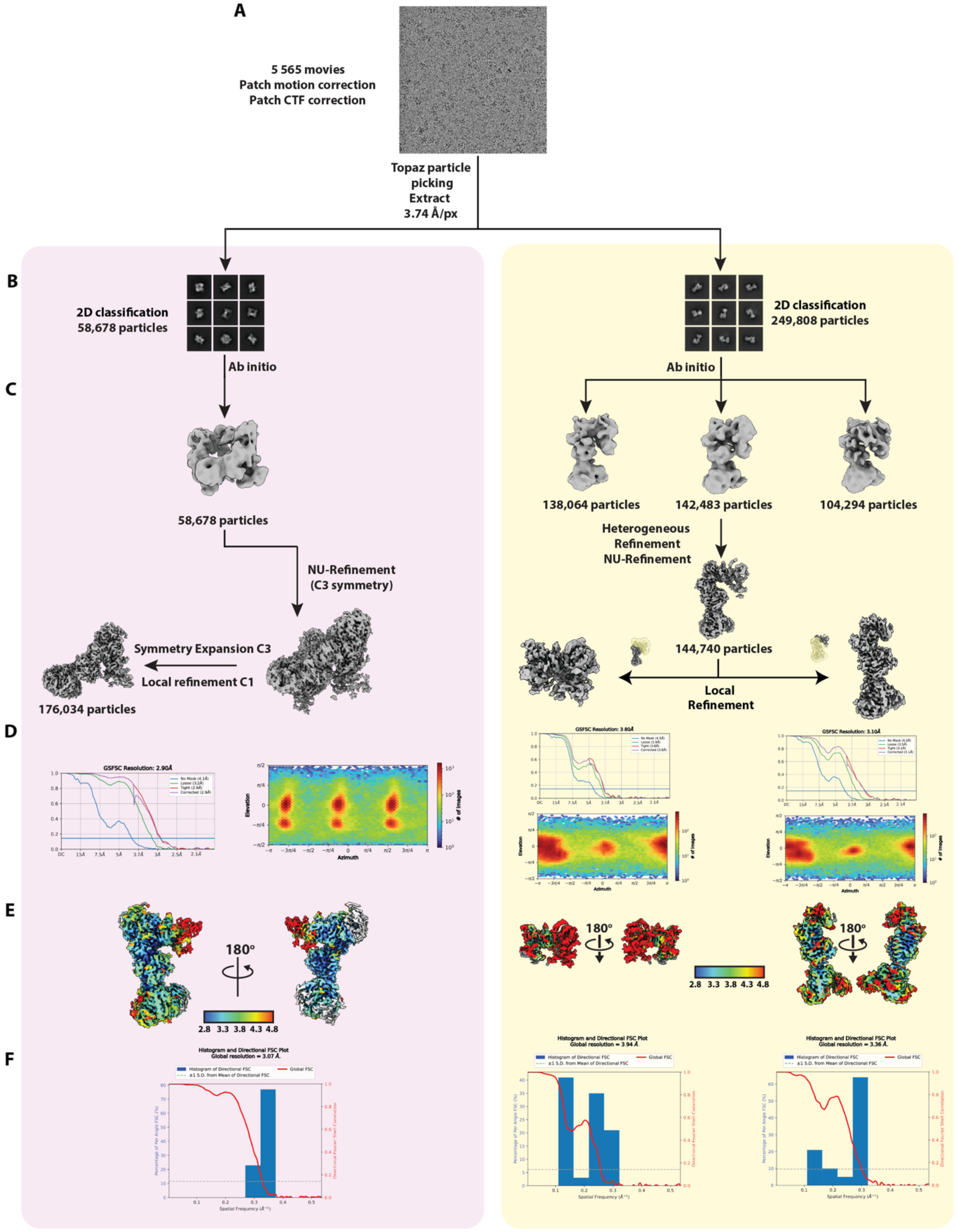

Figure S8. Cryo-EM workflow for LRRK2^{RCKW}(WT):GZD-824

Representative micrograph **(A)**, 2D class averages **(B)**, data processing strategy **(C)**, FSC plots and Euler angle distributions **(D),** local resolution maps **(E)**, and 3D FSC plot **(F)** for LRRK2^{RCKW}(WT):GZD-824 trimer (pink panel) and monomer (yellow panel).

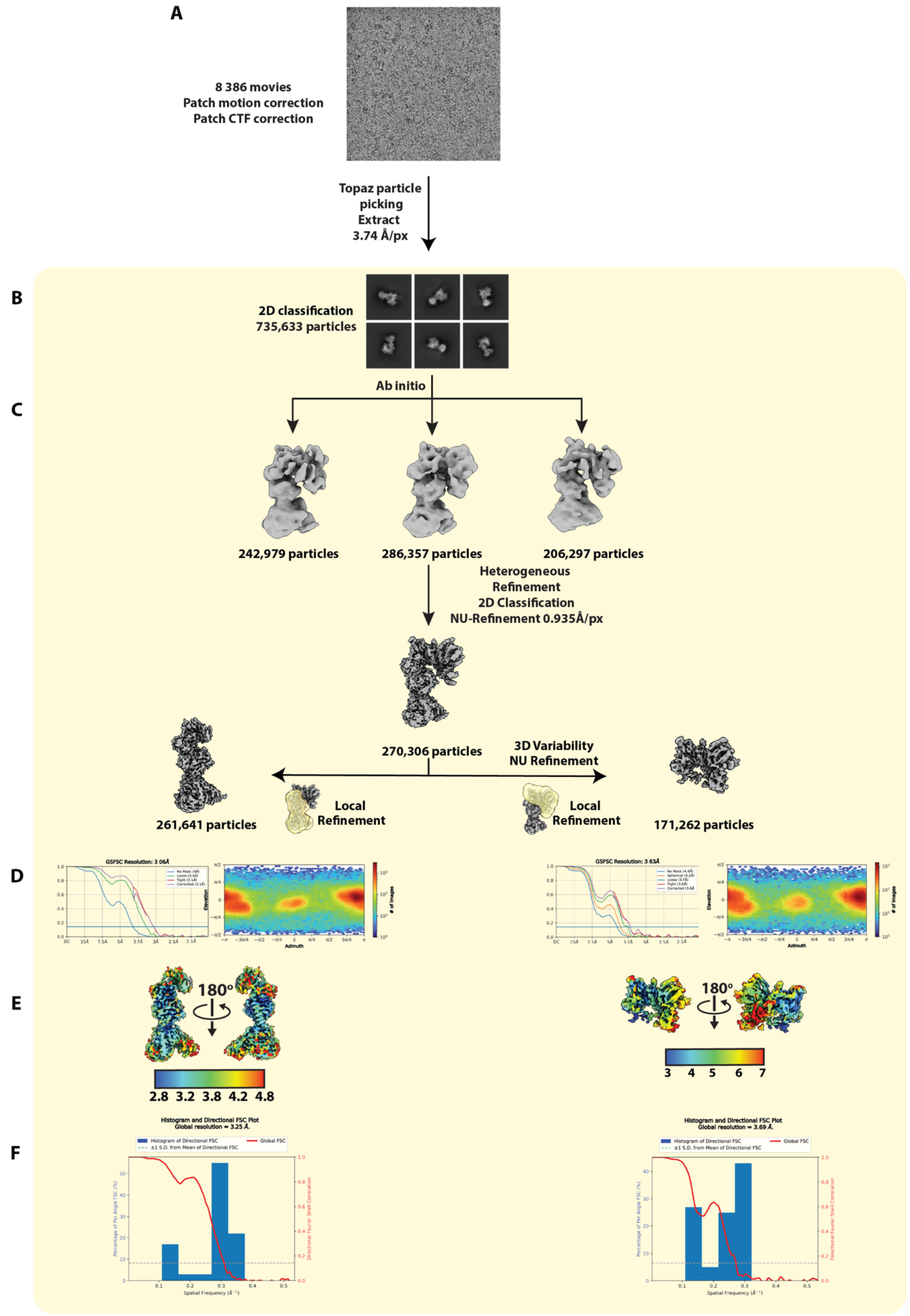

Figure S9. Cryo-EM workflow for LRRK2^{RCKW}(I2020T):GZD-824

Representative micrograph **(A)**, 2D class averages **(B)**, data processing strategy **(C)**, FSC plots and Euler angle distributions **(D),** local resolution maps **(E)**, and 3D FSC plot **(F)** for LRRK2^{RCKW}(I2020T):GZD-824 monomer.

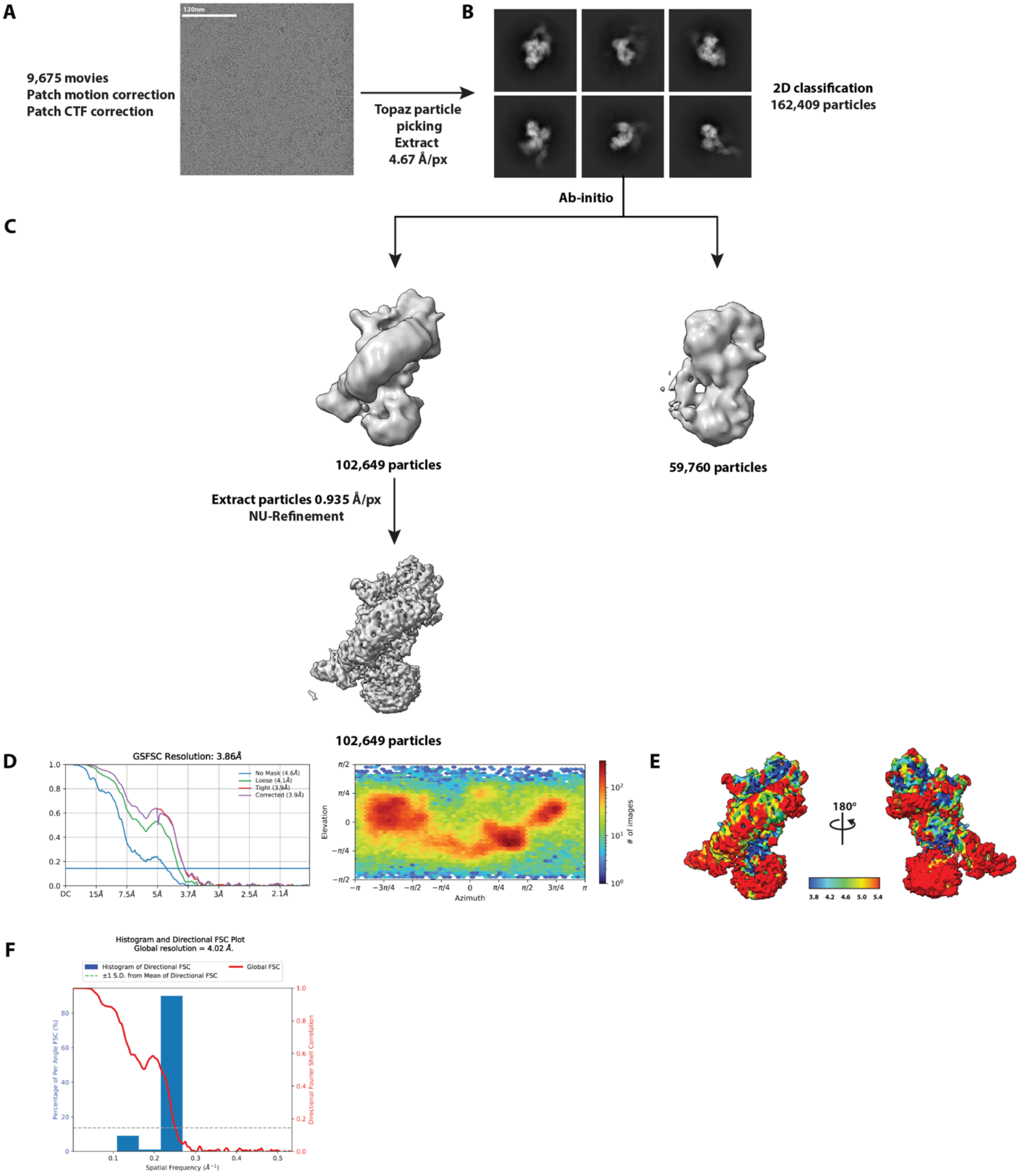

Figure S10. Cryo-EM workflow for LRRK2(I2020T):MLi-2

Representative micrograph **(A)**, 2D class averages **(B),** data processing strategy **(C)**, FSC plots and Euler angle distributions **(D),** local resolution maps **(E)**, and 3D FSC plot **(F)** for LRRK2(I2020T):MLi-2.

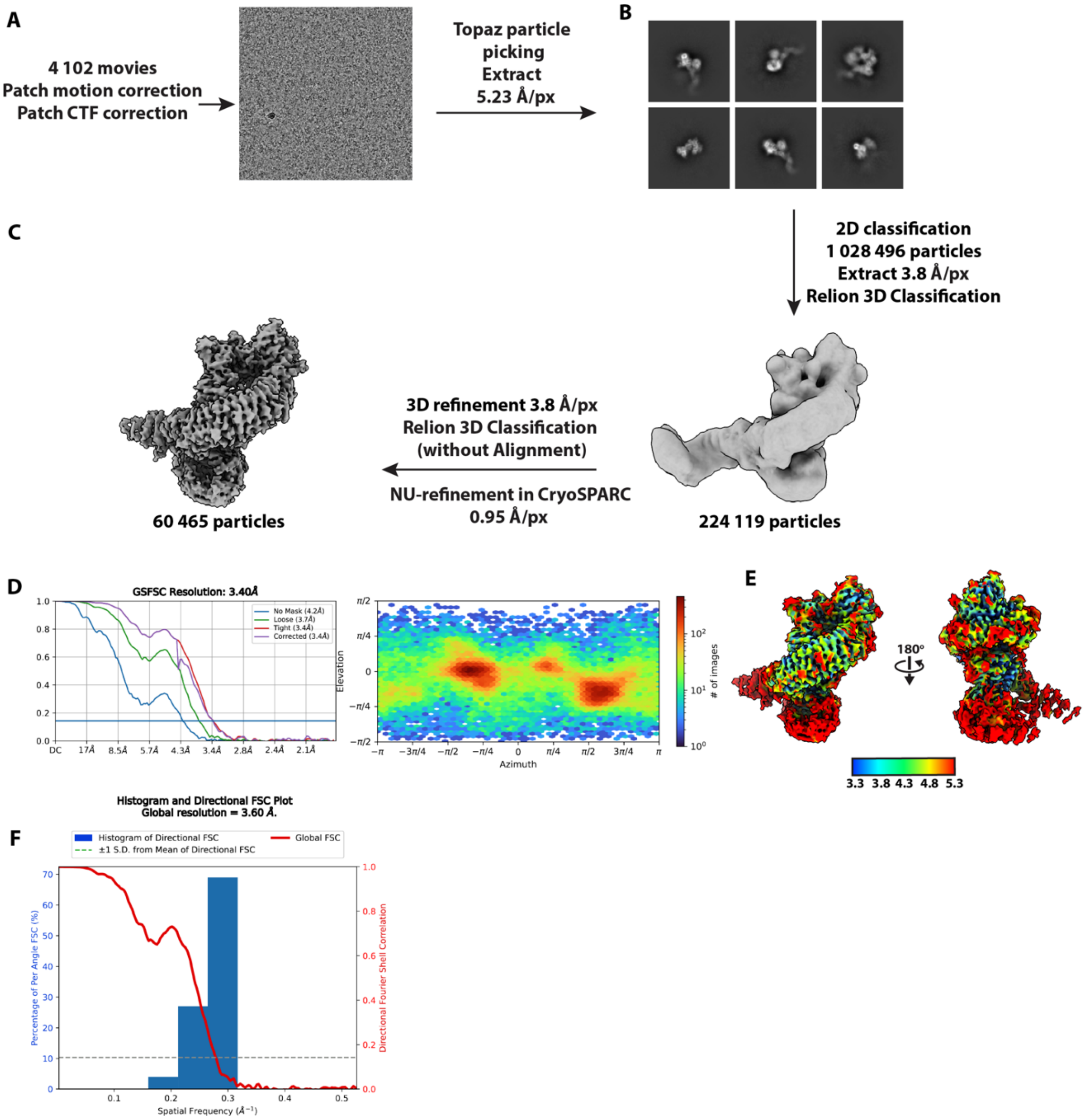

Figure S11. Cryo-EM workflow for LRRK2(I2020T):GZD-824

Representative micrograph **(A),** 2D class averages **(B)**, data processing strategy **(C)**, FSC plots **(D),** local resolution maps **(E)**, and 3D FSC plot **(F)** for LRRK2(I2020T):GZD-824.

Movie S1. Cryo-EM structures of LRRK2^{RCKW} bound to MLi-2 and GZD-824

The movie shows the cryo-EM maps and models for LRRK2RCKW bound to the type-I inhibitor MLi-2 or the type-II inhibitor GZD-824 and highlights how the major structural features of the kinase differ between them.

Table S1. Cryo-EM data collection, refinement, and validation statistics

	LRRK2^{RCKW}(WT): MLi-2:E11 DARPin (tetramer) (EMDB-41709) (PDB 8TXZ)	LRRK2^{RCKW}(G2019S): MLi-2:E11 DARPin (tetramer) (EMDB-41754) (PDB 8TZC)	LRRK2^{RCKW}(I2020T): MLi-2:E11 DARPin (tetramer) (EMDB-41758) (PDB 8TZG)
**Data collection and processing**			
Magnification	105000	130000	130000
Voltage (kV)	300	300	300
Electron exposure (e–/Å^2)	57	55	55
Defocus range (μm)	-1.0 to -3.0	-1.0 to -3.0	-1.0 to -3.0
Pixel size (Å)	0.822	0.935	0.935
Symmetry imposed	D2	D2	D2
Initial particle images (no.)	574 664	548 873	694 336
Final particle images (no.)	584 196	965 742	380 487
Map resolution (Å)	3.05	2.74	2.74
FSC threshold	0.143	0.143	0.143
Map resolution range (Å)	2.8 – 3.6	2.4 - 4.0	2.5-4.1
**Refinement**			
Initial model used (PDB code)	6VP7	6VP7	6VP7
Model resolution (Å)	3.05	2.74	2.74
FSC threshold	0.143	0.143	0.143
Model composition			
Non-hydrogen atoms	6261	7623	7095
Protein residues	814	998	907
Nucleotide	1	1	1
Ligand	1	1	1
Water	1	1	1
B factors (Å^2)			
Protein	42.37	93.28	47.54
Nucleotide	212.72	132.81	121.15
Ligand	23.59	78.41	34.58
Water	12.48	70.18	25.11
R.m.s. deviations			
Bond lengths (Å)	0.019	0.008	0.003
Bond angles (°)	1.312	1.034	0.701
Validation			
MolProbity score	2.30	2.21	1.83
Clashscore	8.85	7.97	8.06
Ramachandran plot			
Favored (%)	93.92	93.74	94.31
Allowed (%)	5.96	6.26	5.58
Disallowed (%)	0.12	0.00	0.12

Table S1. Cryo-EM data collection, refinement, and validation statistics

	LRRK2^{RCKW}(G2019S) :GZD-824:E11 DARPin[a] (monomer) Composite map (EMDB-41728) (PDB 8TYQ)	LRRK2^{RCKW}(G2019S) :GZD-824:E11 DARPin[a] (monomer) Consensus refinement (EMDB-41798)	LRRK2^{RCKW}(G2019S) :GZD-824:E11 DARPin[a] (monomer) Focused refinement Kinase-WD40 (EMDB-41799)	LRRK2^{RCKW}(G2019S) :GZD-824 E11 DARPin[a] (monomer) Focused refinement ROC-COR (EMDB-41797)
**Data collection and processing**				
Magnification	130000	130000	130000	130000
Voltage (kV)	300	300	300	300
Electron exposure (e–/Å^2)	55	55	55	55
Defocus range (μm)	-1.0 to -3.0	-1.0 to -3.0	-1.0 to -3.0	-1.0 to -3.0
Pixel size (Å)	0.935	0.935	0.935	0.935
Symmetry imposed	C1	C1	C1	C1
Initial particle images (no.)	759,293	759,293	759,293	759,293
Final particle images (no.)	261,641/169,878	261,641	261,641	169,878
Map resolution (Å)	--	3.10	2.99	3.50
FSC threshold	--	0.143	0.143	0.143
Map resolution range (Å)	2.7.-7	2.7-6	2.7-4.2	3-7
**Refinement**				
Initial model used	6VP7	--	--	--
(PDB code)		--	--	--
Model resolution	--	--	--	--
(Å)	--	--	--	--
FSC threshold				
Model composition				
Non-hydrogen	8523	--	--	--
atoms	1068	--	--	--
Protein residues	0	--	--	--
Nucleotide	1	--	--	--
Ligands				
B factors (Å^2)				
Protein	171.59	--	--	--
Nucleotide	--	--	--	--
Ligand	48.03	--	--	--
R.m.s. deviations				
Bond lengths (Å)	0.007	--	--	--
Bond angles (°)	0.904	--	--	--
Validation				
MolProbity score	1.72	--	--	--
Clashscore	10.14	--	--	--
Ramachandran plot				
Favored (%)	96.86	--	--	--
Allowed (%)	3.16	--	--	--
Disallowed (%)	0.00	--	--	--

[a] composite map

Table S1. Cryo-EM data collection, refinement, and validation statistics

	LRRK2^{RCKW}(I2020T): GZD-824:E11 DARPin (monomer) Composite map (EMDB- 41753) (PDB 8TZB)	LRRK2^{RCKW}(I2020T): GZD-824:E11 DARPin[a] (monomer) Consensus refinement (EMDB- 41802)	LRRK2^{RCKW}(I2020T): GZD-824:E11 DARPin[a] (monomer) Focused refinement Kinase-WD40 (EMDB-41794)	LRRK2^{RCKW}(I2020T): GZD-824:E11 DARPin[a] (monomer) Focused refinement ROC-COR (EMDB-41795)
**Data collection and processing**				
Magnification	130000	130000	130000	130000
Voltage (kV)	300	300	300	300
Electron exposure (e–/Å^2)	55	55	55	55
Defocus range (μm)	-1.0 to -3.0	-1.0 to -3.0	-1.0 to -3.0	-1.0 to -3.0
Pixel size (Å)	0.935	0.935	0.935	0.935
Symmetry imposed	C1	C1	C1	C1
Initial particle images (no.)	735,633	735,633	735,633	735,633
Final particle images (no.)	261,641/171,262	270,306	261,641	171,262
Map resolution (Å)	--	3.22	3.06	3.63
FSC threshold	--	0.143	0.143	0.143
Map resolution range (Å)	--	2.8-7	2.8-4.4	3-7
**Refinement**				
Initial model used	6VP7	--	--	--
(PDB code)		--	--	--
Model resolution	--	--	--	--
(Å)	--	--	--	--
FSC threshold				
Model composition				
Non-hydrogen	7499	--	--	--
atoms	1012	--	--	--
Protein residues	0	--	--	--
Nucleotide	1			
Ligands				
B factors (Å^2)				
Protein	145.4	--	--	--
Nucleotide	--	--	--	--
Ligand	33.66	--	--	--
R.m.s. deviations				
Bond lengths (Å)	0.004	--	--	--
Bond angles (°)	0.594	--	--	--
Validation				
MolProbity score	1.94	--	--	--
Clashscore	10.46	--	--	--
Ramachandran plot				
Favored (%)	93.94	--	--	--
Allowed (%)	5.96	--	--	--
Disallowed (%)	0.11	--	--	--

[a] composite map

Table S1. Cryo-EM data collection, refinement, and validation statistics

	FL(I2020T):<br>MLi-2:E11 DARPin<br>(monomer)<br>(EMDB-41759)<br>(PDB 8TZH)	FL(I2020T):<br>GZD-824: E11 DARPin<br>(monomer)<br>(EMDB-41757)<br>(PDB 8TZF)	LRRK2^{RCKW}(WT):<br>GZD-824:E11 DARPin<br>(trimer)<br>(EMDB-41756)<br>(PDB 8TZE)
**Data collection and processing**			
Magnification	130000	150000	130000
Voltage (kV)	300	200	300
Electron exposure (e–/Å^2)	50	55	55
Defocus range (μm)	-1.0 to -3.0	-1.0 to -3.0	-1.0 to -3.0
Pixel size (Å)	0.935	0.95	0.935
Symmetry imposed	C1	C1	C3
Initial particle images (no.)	162,409	224,119	58,678
Final particle images (no.)	102,649	60,465	176.034
Map resolution (Å)	3.9	3.4	2.90
FSC threshold	0.143	0.143	0.143
Map resolution range (Å)	3.8 -5.0	3.3-5.3	2.8-4.5
**Refinement**			
Initial model used (PDB code)	7LHW	7LHW	6VP7
Model resolution (Å)	3.9	3.4	2.90
FSC threshold	0.143	0.143	0.143
Model composition			
Non-hydrogen atoms	11,593	12,156	6251
Protein residues	1,606	1,671	787
Nucleotide	1	1	0
Ligands	1	1	1
B factors (Å^2)			
Protein	96.31	174.38	121.98
Nucleotide	102.80	87.23	--
Ligand	82.68	109	45.07
R.m.s. deviations			
Bond lengths (Å)	0.003	0.003	0.007
Bond angles (°)	0.791	0.557	1.096
Validation			
MolProbity score	2.18	1.99	2.42
Clashscore	15.15	9.96	9.49
Ramachandran plot			
Favored (%)	91.79	92.4	93.54
Allowed (%)	8.21	7.60	6.32
Disallowed (%)	0.00	0.00	0.13
